# Environmental monitoring using next generation sequencing: rapid identification of macroinvertebrate bioindicator species

**DOI:** 10.1186/1742-9994-10-45

**Published:** 2013-08-07

**Authors:** Melissa E Carew, Vincent J Pettigrove, Leon Metzeling, Ary A Hoffmann

**Affiliations:** 1 Department of Zoology, Victorian Centre for Aquatic Pollution Identification and Management (CAPIM), The University of Melbourne, Victoria 3010, Australia; 2 EPA Victoria, Ernest Jones Drive, Macleod, Victoria 3085, Australia; 3 Department of Genetics, The University of Melbourne, Victoria 3010, Australia

**Keywords:** Invertebrates, Barcoding, Bioassessment, 454 pyrosequencing, Chironomidae

## Abstract

**Introduction:**

Invertebrate communities are central to many environmental monitoring programs. In freshwater ecosystems, aquatic macroinvertebrates are collected, identified and then used to infer ecosystem condition. Yet the key step of species identification is often not taken, as it requires a high level of taxonomic expertise, which is lacking in most organizations, or species cannot be identified as they are morphologically cryptic or represent little known groups. Identifying species using DNA sequences can overcome many of these issues; with the power of next generation sequencing (NGS), using DNA sequences for routine monitoring becomes feasible.

**Results:**

In this study, we test if NGS can be used to identify species from field-collected samples in an important bioindicator group, the Chironomidae. We show that *Cytochrome oxidase I* (COI) and *Cytochrome B* (CytB) sequences provide accurate DNA barcodes for chironomid species. We then develop a NGS analysis pipeline to identifying species using megablast searches of high quality sequences generated using 454 pyrosequencing against comprehensive reference libraries of Sanger-sequenced voucher specimens. We find that 454 generated COI sequences successfully identified up to 96% of species in samples, but this increased up to 99% when combined with CytB sequences. Accurate identification depends on having at least five sequences for a species; below this level species not expected in samples were detected. Incorrect incorporation of some multiplex identifiers (MID’s) used to tag samples was a likely cause, and most errors could be detected when using MID tags on forward and reverse primers. We also found a strong quantitative relationship between the number of 454 sequences and individuals showing that it may be possible to estimate the abundance of species from 454 pyrosequencing data.

**Conclusions:**

Next generation sequencing using two genes was successful for identifying chironomid species. However, when detecting species from 454 pyrosequencing data sets it was critical to include known individuals for quality control and to establish thresholds for detecting species. The NGS approach developed here can lead to routine species-level diagnostic monitoring of aquatic ecosystems.

## Introduction

Invertebrates represent a key indicator group for monitoring environmental change in many different ecosystems e.g. [1-4]. Invertebrate communities are useful for assessing ecosystem health, as they are common and widespread, with high species diversity and varying sensitivity to environmental disturbances [5]. While considerable effort has gone into developing protocols that utilize invertebrate communities for environmental monitoring, particularly for monitoring freshwater ecosystems [6], a major challenge has been identification to the ‘species’ level. Few species are easily recognized. For most a high level of taxonomic expertise is required, which is more difficult when traditional keys or other reference material is of variable quality or lacking. As a result, identification error rates are significantly higher than for species than family level identifications, impacting quality assurance [7,8]. Furthermore, some specimens cannot be identified beyond higher taxonomic levels as they are morphologically immature, cryptic or represent little known groups [9]. As a result, cruder levels of identification are often used for monitoring. Even though identification at higher taxonomic levels, such as families, can be effective at broader regional or catchment scales to examine the magnitude of impacts or to classify sites, it can miss impacts or changes at smaller scales [10]. It is also less likely to be diagnostic of the specific factors impacting an ecosystem, as genera and species within the same families vary in their responses to pollutants and environmental characteristics [11-13]. For this reason, there has been a move towards generating species and genus level responses to pollutants in some regions [14-17]. However, for diagnostic monitoring that uses the responses of species or genera to be more widely adopted, species identification needs to be more cost effective, rapid and accurate.

DNA sequencing, including ‘DNA barcoding’, can overcome the issues associated with morphological identification and can offer an alternative for making routine species level identifications. DNA sequences can be easily obtained, analysed and interpreted and, with few exceptions, are highly accurate for identifying invertebrate species [18-23]. DNA sequencing certain gene regions has proven useful for understanding species diversity in many taxonomically difficult or poorly studies groups e.g. [24-27] and is often included as part of integrated taxonomic studies e.g. [28-32]. DNA-based species identification can detect more species with greater accuracy than traditional morphological methods for environmental monitoring [8]. However, until recently it has not been feasible to use sequencing for routine monitoring. Even with automated extraction, PCR and sequencing, species would need to be individually sorted which is both laborious and expensive [9,33].

Next generation sequencing (NGS) has potential to be used for routine environmental monitoring as, in a single instrument run, multiple species in many samples can be simultaneously sequenced, reducing the time and cost involved in sample processing [9,33]. Currently, 454 pyrosequencing has been the most widely applied NGS technique for identifying species, as it produces the longer sequences needed for accurate identification compared to other NGS platforms [34]. Most studies employing 454 pyrosequencing in environmental monitoring have focused on method development and testing specific taxonomic groups or environmental samples, such as those from estuarine, marine or rainforest habitats e.g. [33,35-39]. However, continued effort is needed to test NGS to establish if one or more barcoding sequences are adequate for identifying species, and also to determine the detection limits for species in mixed samples along with potential error rates. Furthermore, there is a need for simple analysis pipelines to deal with NGS data that use new bioinformatic tools and software [33,40].

Over the past decade, we have been examining pollution responses and testing DNA sequences for species identification in the Chironomidae [12,13,41-44]. Chironomids are a speciose group consisting of taxa that vary in their responses to pollution and other environmental characteristics, and they are an important biological indicator group for monitoring, assessing and classifying aquatic environments [45-47]. They are particularly useful as indicators of aquatic pollution in urban areas, because they can dominate benthic urban macroinvertebrate fauna in these areas, representing up > 50% of the aquatic insect species collected in benthic surveys [46,48-50]. In field surveys and field based microcosm experiments, chironomid species are diagnostic of particular types of pollution and environmental characteristics [13,41,43,44,51-53]. Through validating field surveys with field based microcosm experiments, we have begun to characterise the distribution of many local chironomid species and their sensitivity to sediment pollution and other environmental characteristics. While taxonomic keys for chironomid identification are available e.g. [54,55], many genera consist of morphologically cryptic species and many species remain undescribed. Where species level identification is possible, it typically requires slide mounting and considerable taxonomic expertise. However, DNA sequences, primarily involving the mitochondrial *Cytochrome oxidase I* (COI) DNA barcode region, are effective for broadly identifying chironomid species [23,56-58].

In this study we develop 454 pyrosequencing for identifying chironomid species from field collected samples. We test whether 454 pyrosequencing of two gene regions commonly used for molecular species identification – mitochondrial COI and *Cytochrome B* (CytB) – can accurately reflect the composition of chironomid species at ten field sites. We first identify species from the sites individually then pool samples for 454 pyrosequencing. A simple pipeline is presented for running and analysing the data from such environmental samples.

## Results

### Individual species identification

**Figure 1:**
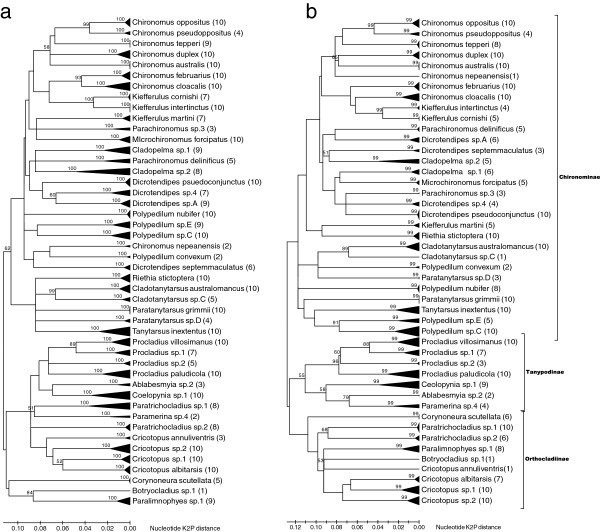
**Bootstrapped Kirma-2**-**parameter trees examining the genetic distance between the species found in this study.** Neighbour joining trees are based on the 46 chironomid species that occurred at the ten field sites for two gene regions **a)** COI **b)** CytB used in this study. Both trees are construct using the same regions used to identify species in the 454 pyrosequencing experiments (395 bps for COI and 343 bps for CytB) and show the level of intraspecific variation (represented by black triangles) based on sequences from up to ten individuals pre species (the number of individuals is given in parentheses) from our DNA reference libraries.

Identification of chironomid samples from the ten field sites indicated 46 chironomid species from three subfamilies (Table 1). Diversity of species ranged from 7 to 14 per site, identified from 32 to 167 individuals collected per site, with a total of 768 individuals collected overall. While 26 species could be identified, the remaining 20 species represented new or known species that could not be identified using only larval keys. These species are denoted as sp.‘x’. Neighbour joining trees for COI and CytB based on up to ten sequences per species for the shorter ‘454 sized’ amplicons showed all species formed distinct groups and these groups were supported by high bootstraps (Figure 1). Mean intraspecific nucleotide variation within species ranged from 0–4.2% for COI and 0–4.4% for CytB, while mean inter-specific variation ranged from 7–34.1% for CytB and 8.7-34.1% for COI, also indicating that the 454 COI and CytB amplicons were suitable for separating species. GenBank accession numbers for these sequences are given in Additional file 1: Table S1.

**Table 1:** Species collected at field site as determined by individual identification and 454 pyrosequencing

**Species**	**Field sites**																			
	**BR08**		**DB09**		**GC09**		**HW09**		**LE09**		**MC09**		**ME09**		**RL09**		**SK09**		**UK09**	
	**n**	**(reads)**	**n**	**(reads)**	**n**	**(reads)**	**n**	**(reads)**	**n**	**(reads)**	**n**	**(reads)**	**n**	**(reads)**	**n**	**(reads)**	**n**	**(reads)**	**n**	**(reads)**
**Chironominae**																				
*Chironomus australis*							100	(5318)	1	(318)	**0**	**(2)**	18	(2370)	1	(394)	1	(8)	71	(5519)
*Chironomus cloacalis*							18	(998)	11	(1580)			1	(79)	1	(23)			5	(511)
*Chironomus duplex*							6	(405)	3	(149)			1	(253)	3	(225)			6	(2327)
*Chironomus februarius*							22	(1104)	6	(821)					20	(861)		(1)	2	(82)
*Chironomus nepeanensis*																			1	(298)
*Chironomus oppositus*	3	(35)	2	(603)	1	(934)			57	(6197)	2	(815)			22	(780)	32	(4803)	**1**	**(0)**
*Chironomus pseudoppositus*															3	(96)				
*Chironomus tepperi*									3	(166)					1	(6)				
*Cladopelma* sp.1									**0**	**(4)**	2	(869)							1	(10)
*Cladopelma* sp.2															8	(292)				
*Cladotanytarsus australomancus*			1	(16)							14	(4239)								
*Cladotanytarsus* sp.C	1	(7)																		
*Dicrotendipes pseudoconjunctus*			4	(999)			3	(100)	6	(479)	1	(1064)	21	(4724)	6	(198)	1	(579)		
*Dicrotendipes septemmaculatus*			**0**	**(1)**			1	(17)												
*Dicrotendipes* sp.4															1	(18)	2	(895)		
*Dicrotendipes* sp.A					2	(1877)														
*Kiefferulus cornishi*					1	(686)							1	(29)			4	(516)	4	(75)
*Kiefferulus intertinctus*			7	(581)	3	(1171)	1	(30)	2	(32)			1	(177)			4	(1737)	1	(236)
*Kiefferulus martini*									1	(168)					1	(18)				
*Microchironomus forcipatus*															1	(7)				
*Parachironomus delinificus*	1	(430)							1	(10)										
*Parachironomus* sp.3	3	(324)																		
*Paratanytarsus grimmii*					1	(308)			3	(264)							5	(1483)		
*Paratanytarsus* sp.D													1	(7)						
*Polypedilum convexum*	3	(332)																		
*Polypedilum nubifer*							9	(401)											1	(63)
*Polypedilum* sp.C			43	(2367)			**0**	**(2)**			2	(929)								
*Polypedilum* sp.E											1	(284)							1	(9)
*Riethia stictoptera*	1	(913)	6	(905)																
*Tanytarsus inextentus*			1	(22)											1	(5)				
Tanypodinae																				
*Ablabesmyia* sp.2	1	(46)																		
*Coelopynia* sp.1	1	(26)							**0**	**(1)**										
*Paramerina* sp.4			1	(74)																
*Procladius paludicola*	11	(1702)	9	(349)			1	(18)			**0**	**(1)**							2	(95)
*Procladius* sp.1			4	(1382)																
*Procladius* sp.2	39	(4609)			**0**	**(4)**														
*Procladius villosimanus*							5	(664)	2	(285)			2	(1392)	30	(6660)				
Orthocladiinae																				
*Botryocladius* sp.1	1	(96)																		
*Corynoneura scutellata*													2	(16)			1	(3)		
*Cricotopus albitarsis*					4	(156)														
*Cricotopus annuliventris*													1	(27)						
*Cricotopus* sp.1					1	(3)														
*Cricotopus* sp.2	4	(31)			19	(3930)					6	(1009)								
*Paralimnophyes* sp.1									2	(69)			2	(27)					**0**	**(2)**
*Paratrichocladius* sp.1							1	(6)												
*Paratrichocladius* sp.2																	1	(211)		
**Total number of individuals**	69		78		32		167		98		28		51		99		50		96	
**Total number of reads**		8551		7299		9069		9063		10543		9212		9101		9583		10236		9227
**Total number of species**	12		10		8		11		13		7		11		14		9		12	

The number of individuals collected in this study at each field site (n) and the number of sequences (reads) from the 454 pyrosequencing experiments representing each species in parentheses. Differences in species detected by individual identification and by 454 pyrosequencing are bolded.

### Species identification of control samples using 454 pyrosequencing

First, we investigated the quality of sequences generated in the 454 pyrosequencing experiment by examining the three control samples containing the two *Chironomus februarius* individuals (Cf1 and Cf2) and a *Chironomus cloacalis* (Cc1) individual in the two biological replicates (runs). Megablast searches of 454 sequences from these individuals against the Sanger generated sequences for the same individual showed >99% of sequences were >98% match to the Sanger generated sequences for COI in both biological replicates. Megablast matches were lower for CytB where >89% of sequences showed a >98% match in the first biological replicate (run) and >93% in the second biological replicate (run). However, when a >97% match was considered for CytB, >98% and >99% of sequences matched the Sanger generated sequence for the same individual for the first and second biological replicate, respectively. Based on these results and the levels of intra- and inter-specific diversity found in our DNA reference database, we identified a ‘species’ if a 454 sequence shared >97% match in megablast searches to a Sanger generated sequence in our DNA reference database.

We then searched the control sequences against our entire DNA reference database. While nearly all sequences generated in the 454 pyrosequencing experiment provided <97% match to the correct species in the DNA reference databases, a small number of sequences were close matches to species absent in the control samples but present in our experiment. These sequences were represented by less than three sequences or <0.07% of sequences generated for each control sample. They appeared to be randomly distributed in relation to run, direction and gene. In sorting sequences from the 454 pyrosequencing experiment, 8% of sequences contained MID combinations that were not used. However, these incorrect MID combinations were almost entirely composed of MID’s used in the experiment.

### Species identification of field collected samples using 454 pyrosequencing

Based on the total number of species found at each of the ten field sites, we had 107 opportunities to detect a species as being present at a field site in our 454 pyrosequencing experiment (Table 1, Additional file 2: Table S2). To determine how well the method performed in detecting species in samples, we examined the ten field sites by gene (COI or CytB), direction (forward or reverse sequences) and run (biological replicate) (Table 2). We found little variation between biological replicates, with the number of sequences generated for a species highly correlated (COI *r* = 0.980; CytB *r* = 0.967). However, the total number of reads between genes was not as strongly correlated (*r* = 0.587) suggesting more variation between genes than biological replicates. *Cytochrome oxidase I* was able to detect more species than CytB, with only four species missed when examining all COI sequences, including both runs and directions, compared to 18 species that were missed by CytB when including both runs and directions. For both genes, more species were detected when both forward and reverse sequences were considered, with an additional one to three species found for COI and four to six species for CytB in each run. However, the best results were achieved when both genes were considered in a run. Only two species were missed in run 1 and one species in run 2, resulting in only a single species not being detected in the entire 454 pyrosequencing experiment.

**Table 2:** Success of species detection using 454 pyrosequencing

**Direction/run/gene**	**Species detected**	**Species missed**	**% Identification success rate**
*Run 1*			
COI forward	100	7	93.46
COI reverse	98	9	91.59
COI both directions	103	4	96.26
*Run2*			
COI forward	98	9	91.59
COI reverse	101	6	94.39
COI both directions	102	5	95.33
*Both runs*			
COI forward	101	6	94.39
COI reverse	102	5	95.33
COI both directions	103	4	96.26
*Run 1*			
CytB forward	78	29	72.90
CytB reverse	79	28	73.83
CB both directions	83	24	77.57
*run2*			
CytB forward	77	30	71.96
CytB reverse	80	27	74.77
CytB both directions	86	21	80.37
*Both runs*			
CytB forward	85	22	79.44
CytB reverse	83	24	77.57
CytB both directions	89	18	83.18
**Both genes for run1**	**105**	**2**	**98.13**
**Both genes for run2**	**106**	**1**	**99.07**
**All runs**/**direction**/**genes combined**	**106**	**1**	**99.07**

Species detected and missed from the ten field sites using 454 pyrosequencing examining each gene in each direction (forward or reverse) and both directions (combining forward and reverse sequences) and combining these across biological replicates (runs). The result for both genes combined in each run (biological replicate 1 and biological replicate 2) and the results for the entire experiment are bolded. Values given are based on 107 opportunities to detect a species across all ten field samples (i.e. the sum of the number of species for each of the 10 field sites).

Similar to our control samples, we also found a low number of sequences producing megablast hits for species that were not present at a field site (Table 1). We had nine hits for species that were not expected, spread across seven of the ten field sites. The number of sequences that produced hits for these nine species was less than five and typically only involved one gene in one direction (Additional file 2: Table S2). As in the control samples, the nine species were present in our study but were not detected in individual identifications for those sites. Without the individual identifications, we would have been unable to distinguish these unexpected sequences from species present at low frequency represented by few 454 sequences. We found three small species represented by a single individual in a sample, and represented by five or fewer 454 sequences. If we used a threshold of greater than five sequences for determining a species presence at a site, we would have missed four species in the entire 454 pyrosequencing experiment. If we used this same threshold when considering both genes for each run, nine species would have been missed in run 1 and five species in run 2. In all cases, the species that were missed by 454 pyrosequencing were only represented by single individuals. Nevertheless >90% of the species in this study were represented by greater than ten sequences (Table 1), meaning that, even with a conservative threshold of ten sequences, the majority of species could be confidently detected.

### Species abundance of field collected samples using 454 pyrosequencing

**Figure 2:**
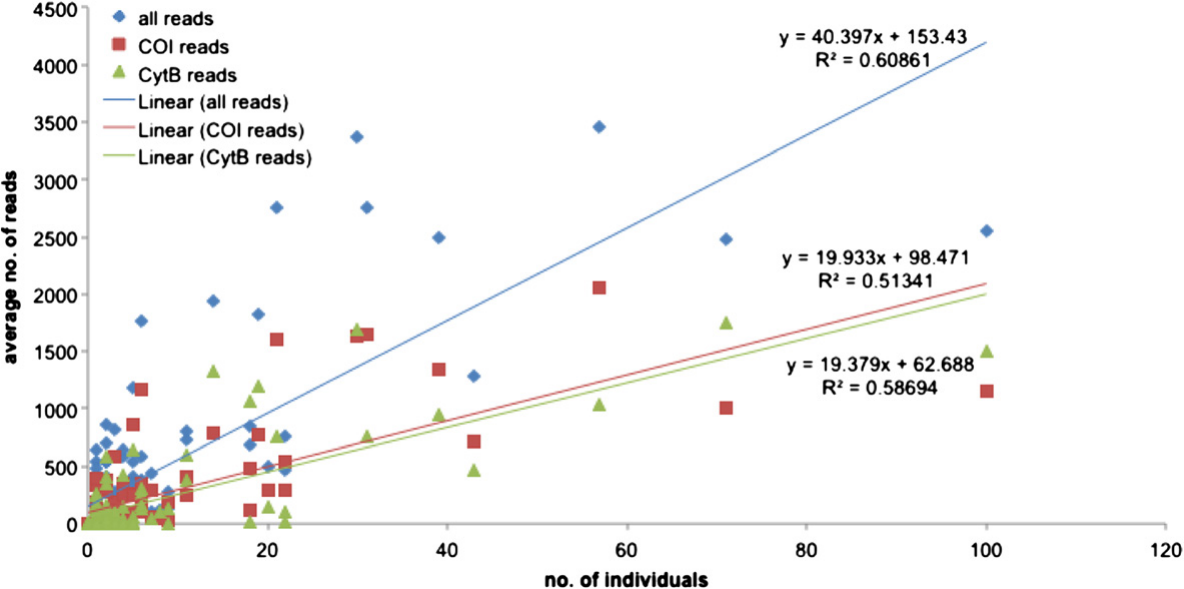
**Relationship between the number of individuals of a species at a site and the average number of 454 sequence reads.** The R^2^ values are shown for ‘all reads’ that combines the data for COI and CytB, and for each gene individually.

**Figure 3:**
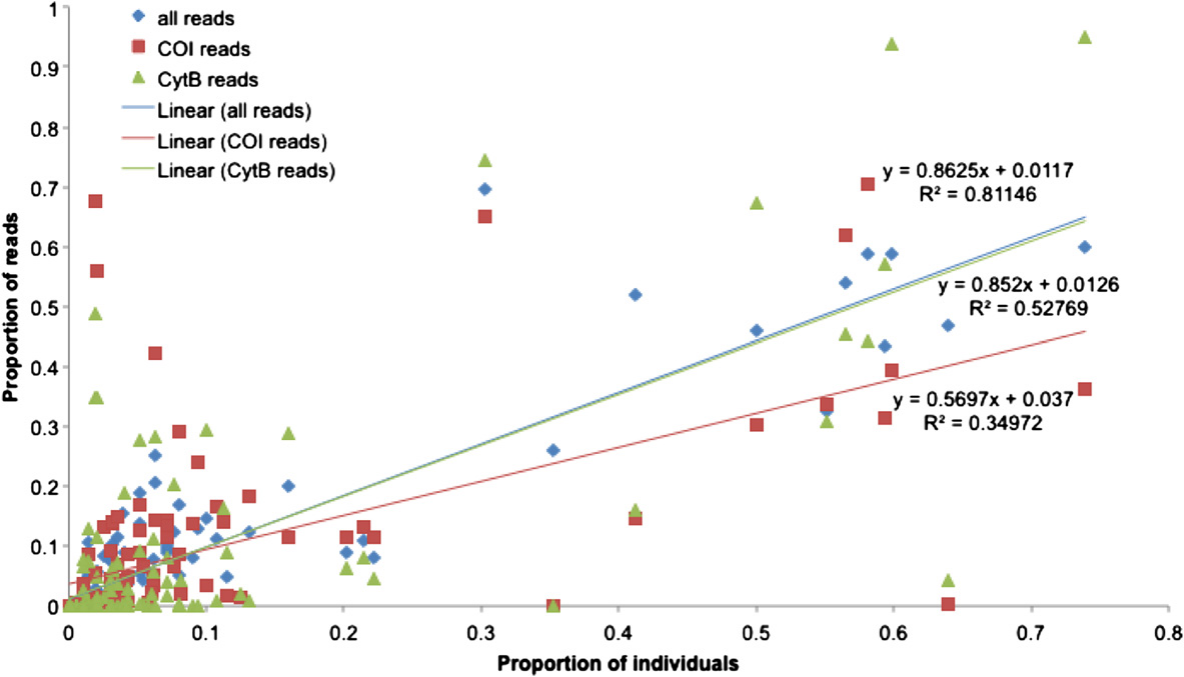
**The relationship between the proportion of individuals per species and the proportion of 454 reads per species per site.** The R^2^ values are shown for ‘all reads’ that combines the data for COI and CytB, and for each gene individually.

**Figure 4:**
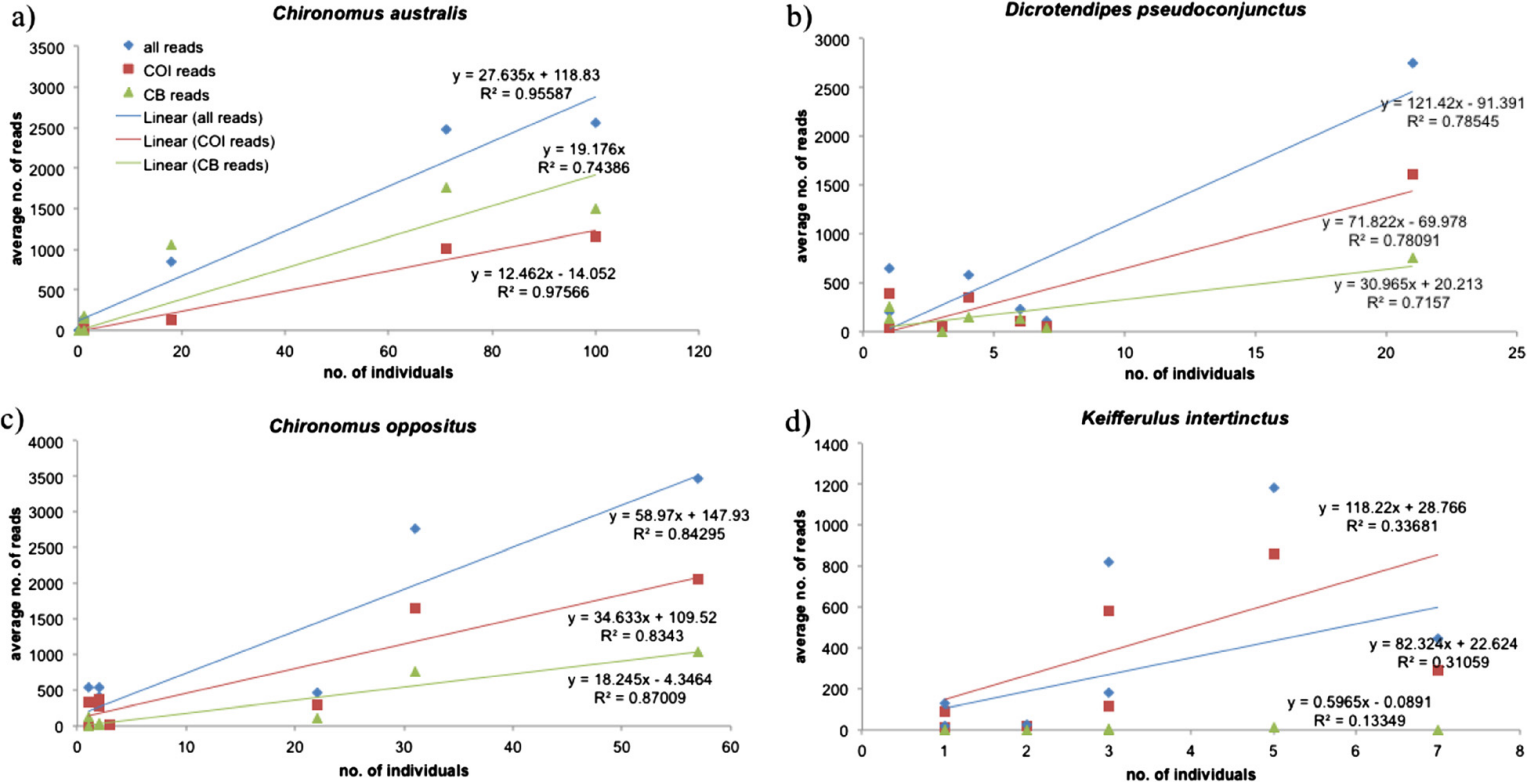
**Relationship between the number of individuals at a site and the average number of 454 sequence reads at a site for four common species a) *Chironomus australis*, b) *Dicrotendipes pseudoconjunctus*, c) *Chironomus oppositus*, and d) *Kiefferulus intertinctus.*** The R^2^ values are shown for ‘all reads’ that combines the data for COI and CytB, and for each gene individually.

The number of individuals of a species and number of reads in a sample were positively associated, suggesting that the number of reads served as a quantitative measure of the abundance of a species at a site; this relationship was evident when reads from both genes were combined and for the individual genes (Figure 2). This relationship was also apparent if proportional data rather than absolute numbers were used (Figure 3). We examined four relatively common species that were present at more than five field sites (Figure 4), and found that at the species level the average number of reads was also strongly related to the number of individuals, except in the case of *Kiefferulus intertinctus* where the CytB primers mostly failed to amplify. For the other species the R^2^ values tended to be higher than in the comparison across all species, perhaps reflecting the fact that conspecific individuals were more likely to be the same size and/or have similar levels of amplification.

## Discussion

Next generation sequencing is capable of identifying species for environmental monitoring in diverse groups like the Chironomidae. Our study showed that almost all species could be successfully identified by megablast searching sequences generated from 454 pyrosequencing for COI and CytB against DNA reference databases of Sanger sequenced voucher species. This approach has been successful for identifying other aquatic invertebrates from the Ephemeroptera and Trichoptera [33], species of nematodes in tropical rainforests [39] and terrestrial invertebrates for biodiversity assessment [59].

Like Hajibabaei *et al*. [33], prior to 454 pyrosequencing we constructed comprehensive DNA reference databases that included all species found in environmental samples to simplify analyses of 454 datasets and subsequent identification of species. We followed a simple filtering approach to remove low quality sequences, and megablast searched all high quality sequences using a ‘best match’ approach, where sequences with a pairwise identity greater than 97% were used for species level identifications. We found this approach to be accurate when compared back to individual identifications, despite the fact that 454 sequences had not been edited to remove errors introduced during PCR or 454 pyrosequencing. While editing sequences would be essential in studies where taxonomic knowledge is low and phylogenetic sorting of sequences into molecular operational taxonomic units (MOTU or OTU) [60] is used to determine diversity e.g. [37,38], our approach suggests that it is not essential when comprehensive DNA reference databases are available. Megablast searching sequences against DNA reference databases also avoids errors in estimating species diversity due to sequencing of products with PCR errors and chimeric sequences which are problematic when assigning OTU’s [38]. While phylogenetic sorting of 454 sequences from environmental samples into OTU’s can still result in identification of species through comparing representative sequences to GenBank for identification [61], DNA barcoding of individual invertebrate species and the development of reference databases makes this approach easier and would be valuable in routine environmental analysis [33].

We trialled two amplicons, COI and CytB, and used amplicons that were longer that those typically used for species identifications with 454 pyrosequencing [33,39,62]. The phylogenetic clustering of species supported by strong bootstraps indicated that the COI and CytB regions selected were able to reliably identify species. High bootstraps and a gap between intra-specific and inter-specific nucleotide diversity showed that the COI and CytB regions we used had performed in a similar way to distinguish species as full COI barcodes e.g. [18,27,63,64]. However, there was evidence of PCR biases present in the 454 dataset. *Cytochrome oxidase I* outperformed CytB for detecting species with 454 pyrosequencing, and the COI primers were capable of amplifying all species, but they failed to detect some species represented by singular small individuals. Hajibabaei *et al*. [33] also found low frequency species could be missed by 454 pyrosequencing of environmental samples of Ephemeroptera and Trichoptera. In contrast, the CytB primers failed to consistently amplify some species and these primers will require redesigning if they are to be used in the future. Yet CytB did detect some species missed by COI, highlighting the potential benefits of using two markers. Porazinska *et al*. [65] also found that using two genes improved the detection level of nematode species from 90 to 97%. In our study, sampling two genes mitigated some of the PCR bias during amplification, which is fundamental to applying PCR-based NGS approaches to species identification in environmental samples [34]. The use of longer amplicons is also likely to have minimized problems associated with “zombie” DNA, such as DNA from dead animals or in gut contents, as this would be degraded and not easily amplified [40,66].

The inclusion of control samples when completing 454 pyrosequencing proved useful for determining the quality of the 454 pyrosequencing run. By comparing two individuals from the same species against an individual from a closely related species, we could establish a threshold for matching sequences directly against our DNA reference database. Such controls could also assist in studies where DNA reference database are not used, as in the case of studies that rely on constructing OTU’s. In these studies, including controls could help determine thresholds for constructing OTU’s, as it enables reliable estimates of 454 pyrosequencing errors. In future studies, it would be useful to include control samples containing known mixtures of species that can provide additional estimates of 454 pyrosequencing errors and PCR biases. Samples with a known constitution might also be used to determine a threshold number of sequences for accepting species presence in a sample.

We found a low number of sequences had blast hits for species not expected to be in a sample based on the individual identifications. Using a threshold five or more sequences, we were unlikely to have included these ‘unexpected’ sequences in our species diversity estimates; however this also meant that we were unable to detect three small rare species present in samples. Exclusion of a few rare taxa is unlikely to cause issues for routine monitoring, particularly as rare taxa are often eliminated in bioassessment analysis [67-69]. Hajibabaei *et al*. [33] also found unexpected sequences in their 454 pyrosequencing run; they attributed these to carry over of DNA from specimens stored in the same preservation media. However, this issue is unlikely to explain our results, because fresh ethanol was used to store field samples. It seems more probable that these sequences originated either as errors in the emulsion PCR or 454 pyrosequencing. Carlsen *et al*. [70] found that sequences could be generated with the wrong MID combination, and they suggested that carry over of low concentrations of unincorporated fusion primers to emulsion PCR might result in mis-tagging of some 454 sequences. Our results are consistent with this explanation rather than first round PCR contamination, as we found 8% of 454 sequences contained incorrect MID combinations but were comprised almost entirely of MID’s that were already used in the experiment. Similarly, all species that were incorrectly identified as being present at a site were already in our experiment. Like Carlsen *et al*. [70], we found that checking both forward and reverse MID tags and eliminating sequences with incorrect reverse MID’s reduced errors in our 454 pyrosequencing experiment. We advocate the addition of MID’s to both ends of PCR amplicons, along with the inclusion of controls. Bidirectional 454 pyrosequencing of multiple genes rather than biological replication also improved detection of species. There was little variation between runs in the two biological replicates in our experiment, supporting previous observations of the robustness of 454 pyrosequencing [71].

A strong quantitative relationship between the 454 sequence reads and species abundance was observed in our dataset, despite samples containing a range of instars and different sized species, which varied from less than 3 mm to up to 13 mm in length. Consistent with Porazinska et al. [65], the frequency of reads did not perfectly mirror the frequency of a species but indicated the relative abundance of a species in a sample, with relationships strongest when comparing the proportion of 454 sequence reads to the proportion of species. Inclusion of multiple genes led to more reliable estimates of the relative abundance of species in samples. When the data from COI and CytB was combined, the relationship of 454 sequences with the number of individuals in a sample tended to strengthen, perhaps through mitigation of PCR biases associated with particular primer sets. These findings suggest that the relative abundance of species could be estimated on a site by site basis, where common species are distinguishable form rare species based on the proportion of reads produced by 454 pyrosequencing.

The inclusion of nuclear genes could further improve the detection of species and their relative abundance. While a number of nuclear loci are already used e.g. [37,38,65], they often have insufficient variation for distinguishing closely related species. Nevertheless, more rapidly evolving nuclear loci, such as the *Carbamoyl*-*phosphate synthetase* region [72], may be useful for separating closely related species that cannot be identified by COI and CytB and also be used for detecting hybridization events. However, the inclusion of nuclear loci will increase the cost of performing identifications based 454 pyrosequencing, and this needs to be weighed against precision required in monitoring programs.

## Conclusions

We found that the NGS approach successfully identified 46 different chironomid species across the ten field sites, including many species that could not be easily identified based on morphology. While many studies have shown the success of DNA methods for identifying chironomid species [23,56-58], this study takes an additional step in demonstrating the feasibility of NGS in routine monitoring of field samples. Previous field-based microcosm experiments and surveys [12,13,17,41,44] have revealed pollution sensitivities and environmental preferences for around half of the species found at the ten field sites. For example species such as *Tanytarsus inextentus*, *Riethia stictoptera* and *Kiefferulus martini* show sensitivity to urban pollution, with the latter two species have also been shown to be pollution sensitive in field-based microcosm experiments [12,13]. Species like *Chironomus duplex* can tolerate high levels of pollution, and others like *Dicrotendipes pseudoconjunctus* are associated with salinity. Chironomid species may also be useful in separating different types of pollutants; species such *Paratanytarsus grimmii* are rare in water bodies where there are high levels of heavy metals and petroleum hydrocarbons occur, but increase in abundance under high nutrient concentrations [12,41,44]. On the other hand, *Procladius villosimanus* declines in response to petroleum hydrocarbons but can be found at field sites with high heavy metal concentrations [41,73]. The next step is to continue to build species tolerance profiles to specific types of pollutants, facilitated by NGS-based identification of bulk samples from field-based microcosms and field site assessments. A combination of species tolerance profiles and NGS across multiple taxa should provide diagnostic assessments of pollutants for routine environmental monitoring.

## Methods

### Reference DNA sequence database

**Figure 5:**
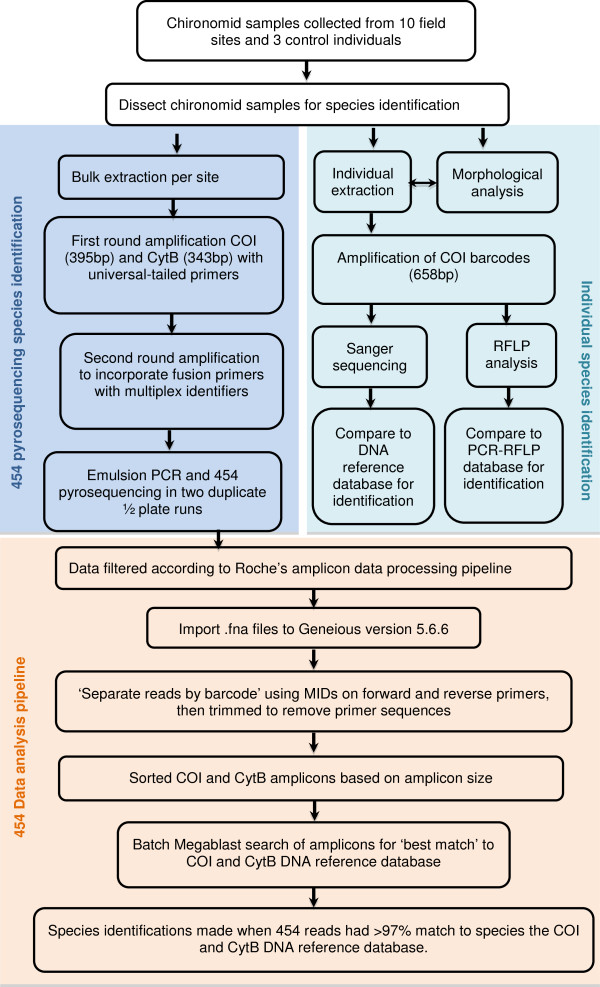
**Experimental design and data analysis pipeline.** The first half of the pipeline (in blue) shows the experimental set up, where species in samples were amplified individually (using morphology, PCR-RFLP and Sanger sequencing) and in bulk using 454 pyrosequencing. The second half of the pipeline (in orange) deals with the analysis of the sequences generated with 454 pyrosequencing.

Reference DNA sequence databases, based on partial sequences from mitochondrial COI and CytB genes, were constructed with multiple individuals from over 120 Chironomidae species collected largely from south-eastern Australia. During construction of the databases, two COI primer combinations were used to amplify 658 bp of the COI gene. For recently processed samples we used the ‘COI barcoding primers’ HCO2189 and LCO1490 (Table 3, Additional file 3: Figure S1a) according to the PCR conditions in Krosch *et al*. [74], while for older samples the primers 911 and 912 were used according to the PCR conditions in Carew *et al*. [75] (Table 3, Figure 5). Two primer combinations were also used to amplify the CytB gene. The primers CB1 and T-N-S1 amplified between 742 bps and 837 bps of the 5’ end of the CytB gene and part of the length variable tRNA serine (Table 3, Additional file 3: Figure S1b). As these primers do not universally amplify CytB in the Chironomidae, we designed a second degenerate primer CB 549 R (Table 3, Additional file 3: Figure S1b), which when used with CB1 amplified 592 bp of the CytB gene. Both CytB fragments were amplified according to the PCR conditions in Carew *et al*. [73]. All PCR products were sequenced in both directions, with sequencing reactions and runs performed by Macrogen (Seoul, Korea). Forward and reverse sequences were aligned and manually edited in Sequencher (version 4.7, Genecodes, Ann Arbor, MI, USA). Consensus sequences for each individual were then exported as concatenated fasta files from Sequencher and imported into Geneious version 5.6.6 [76], where they were used as reference DNA databases to identify species from the 454 pyrosequencing experiment.

**Table 3:** Template specific primers used in this study

**Primer name**	**Sequence (‘5 to 3’)**	**Gene**	**Reference**
HCO2198/ 912	TAAACTTCAGGGTGACCAAAAAATCA	COI	[77]
LCO1490	GGTCAACAAATCATAAAGATATTGG	COI	[77]
COI A for	CCHCGAATAAATAATATAAGWTTYTG	COI	This study
911	TTTCTACAAATCATAAAGATATTGG	COI	[78]
CB1	TATGTTTTACCATGAGGACAAATATC	CytB	[79]
CB322 R	GGRTTDGCDGGRATRAARTTATC	CytB	This study
CB549 R	TTCTACDGTDGCHCCAATTCA	CytB	This study
T-N-S1	TATTTCTTTCTTATGTTTTCAAAAC	CytB	[79]

Primers were used for Sanger sequencing and in 454 pyrosequencing.

### 454 Pyrosequencing design

Chironomid larval samples were collected from ten field sites in 2008–2009 (Table 4, Figure 5). These field sites were selected as they were expected to contain a diversity of chironomid species at different abundance levels based on earlier work. Chironomid larvae were collected using the methods outlined in Carew *et al*. [13], except that larvae were picked on site and placed in 100% ethanol, and stored at 4°C. In the laboratory, samples were sorted and identified to genus. Individuals in each sample were identified to species using either morphological or molecular methods or both depending on the genus. Morphological identifications involved removal of head capsules (and in some cases rear parapods for Tanypodinae) and mounting in Hoyer’s medium. Larval keys by Cranston [54] were used to identify species. In cases where morphological identification was unable to identify species, molecular identifications were performed by dissecting a small amount of tissue from individual chironomids, leaving most of the remaining body for bulk DNA extraction (see below). Molecular identifications were performed using the method outlined in Carew *et al*. [75]. This involved extracting DNA using a Chelex extraction method and amplifying the COI barcode region for either restriction fragment length (RFLP) analysis or sequencing. Profiles generated by RFLP analysis were compared to those found in Carew *et al*. [13] to identify species. Any new or ambiguous RFLP profiles were sequenced to determine if they represent new species or variants of known species.

**Table 4:** **Collection information for Chironomidae samples used in the 454**-**pyrosequencing experiment**

**Site code**	**Site**	**Date**	**Latitude**	**Longitude**
BR08	Barwon River at Pollocksford Rd, Stonehaven, Victoria, Australia	13-Oct-08	−38.15	144.19
DB09	Deep Creek at Bulla Rd, Bulla, Victoria, Australia	7-Oct-09	−37.63	144.80
GC09	Gardiners Creek at High St, Glen Iris, Victoria, Australia	13-Oct-09	−37.89	145.14
HW09	Highlands Wetland Estate, Cragieburn, Victoria, Australia	9-Oct-09	−37.59	144.90
LE09	Lynbrook Estate Wetlands at Lynbrook Boulevard, Lynbrook, Victoria, Australia	6-Oct-09	−38.06	145.25
MC09	Maribyrnong River at Caulder Hwy, Keilor, Victoria, Australia	7-Oct-09	−37.69	144.80
ME09	Brodies Lakes at Greenvale Reservoir Park, Greenvale, Victoria, Australia	12-Oct-09	−37.63	144.89
RL09	Red Leap Reserve, Mill Park, Victoria, Australia	9-Oct-09	−37.67	145.06
SK09	Shankland Wetland, Meadow Heights, Victoria, Australia	12-Oct-09	−37.65	144.91
UK09	Platypus Ponds, Sunbury, Victoria, Australia	7-Oct-09	−37.55	144.74

The remaining tissue of individuals from each site was placed in 1.5 ml microcentrifuge tubes for bulk extraction and PCR, and subsequent 454 pyrosequencing. A Qiagen DNeasy® Tissue Kit (Qiagen, Hilden, Germany) was used to extract total genomic DNA from tubes of chironomid tissues following the manufacturer’s protocol. DNA extraction from some sites required the use of multiple tubes, so after extractions were completed tubes from the same site were mixed thoroughly and aliquots of genomic DNA were pooled into a single tube representing the site. In addition to the ten field sites, we also included three control samples that consisted of two individuals from one species, *Chironomus februarius*, and an individual from a closely related species, *Chironomus cloacalis*. These individuals were Sanger sequenced to generate a consensus sequence and used to assist assigning of sequences to species from the 454 pyrosequencing experiment and to examine the quality of the 454 pyrosequencing data. The 454 pyrosequencing experiment was completed as two duplicate quarter plate runs with each quarter containing a biological replicate. The biological replicates were initiated after the bulk DNA extraction step where we took two aliquots of each DNA extraction, which represented a site/ control. These were used for PCR and 454 pyrosequencing. All steps for each biological replicate were performed independently.

### PCR conditions for 454 pyrosequencing

A two-step PCR process involving a universal tail design was used to obtain amplicons for 454 pyrosequencing. The first PCR involved independently amplifying the two mitochondrial genes from each genomic DNA sample. For this purpose a new PCR primer for each mitochondrial gene was designed to produce amplicons of optimal size (<600 bp) for 454 pyrosequencing (Table 3). These new primers were designed from multi-species alignments (with degenerate bases placed at variable base positions) and verified against a panel of taxonomically divergent chironomid species (data not shown). For COI PCR’s, we used the primer pair COIAfor and LCO1490 which yielded 395 bps of COI sequence, and for CytB PCR’s we used the primer pair CB1 and CB322R which yielded 343 bps of CytB sequence. Each template specific primer contained a universal tail on the 5’ end, which was identical to that on the 3’ end of the 454 specific fusion primers used in the second PCR (see Additional file 4: Table S3). We developed a unique pair of GC rich universal tails (Tail 1 – CAGGACCAGGGTACGGTG and Tail 2 - CGCAGAGAGGCTCCGTG) by substituting bases on ‘Tail C’ and ‘Tail D’ from Blacket *et al*. [80] until they did not form strong hairpins or dimers with the template specific or 454 adaptor sequences. For both COI and CytB, each first round PCR reaction contained 1 μl of DNA template, 17.4 μl molecular biology grade water, 2.5 μl PCR buffer, 1 μl MgCl_2_ (50 mM), 2 μl dNTPs mix (25 mM), 0.5 μl forward primer (10 mM), 0.5 μl reverse primer (10 mM), and 0.1 μl Platinum Taq polymerase (5 U/ml) (Invitrogen, Carlsbad, CA, USA) in a total volume of 25 μl. First round PCR’s were performed using the following conditions: for COI, 94°C for 3 min followed by 20 cycles of 94°C for 20 sec, 48°C for 45 sec, 72°C for 30 sec, then 1 cycle of 72°C for 5 min; for CytB, 94°C for 3 min followed by 20 cycles of 94°C for 20 sec, 45°C for 45 sec, 72°C for 60 sec, then 1 cycle of 72°C for 5 min. Amplifications were performed as six replicates for COI and CytB per site. All PCR products were checked by agarose gel electrophoresis. The six PCR replicates were pooled and cleaned with a PureLink® PCR purification kit (Invitrogen, Carlsbad, CA, USA) using the HC buffer which eliminates dimers up to 300 bps.

Second round PCR’s were performed for each gene and site separately for each reaction using 1 μl of a 1 in 10 dilution of the first round PCR products, 12 μl molecular biology grade water, 15 μl BIO-X-ACT short mix (Bioline, London, England), 1 μl forward primer (10 mM), and 1 μl reverse primer (10 mM) (Table 3). PCR conditions were as follows: 94°C for 5 min followed by 10 cycles of 94°C for 40 sec, 60°C for 40 sec, 72°C for 60 sec, then 1 cycle of 72°C for 5 min. All PCR products were checked by agarose gel electrophoresis, then cleaned with a PureLink® PCR purification kit (Invitrogen, Carlsbad, CA, USA) using the HC buffer and quantified with a Qubit Fluorometer (Life Technologies, Carlsbad, CA, USA). PCR products were then combined in equal molar ratios in two separate tubes representing each biological replicate.

Emulsion PCR, titration and the two quarter 454 pyrosequencing runs were done on a GS-FLX + and were performed following the manufacturer’s protocols for titanium series reagents (Roche Applied Science, Basel, Switzerland) by The Ramaciotti Centre for Gene Function Analysis at the University of New South Wales, Australia. The resulting libraries of sequences were filtered according to amplicon data processing pipeline in the manufacturer’s manual (Roche Applied Science Basel, Switzerland).

### Analysis of 454 pyrosequencing data

All 454 pyrosequencing data was deposited on NCBI Sequence Read Archive under the following accession numbers [SRR867649, SRR867650, SRR869571-SRR869595]. Filtered datasets were imported into Geneious version 5.6.6 [76] and separated by the 3’ multiplex identifiers (MID) (Figure 5). We then trimmed to remove reverse adaptors and reverse complimented sequences and sorted for the expected 5’ MID. Amplicons without correct MID pairs were omitted. Amplicons from COI and CytB were then separated based on amplicon length and trimmed to remove primer sequences. Batch megablast searches for the sequences from each gene from each sample using sequences from one direction at a time were performed against the reference DNA databases for COI and CytB. Megablast searches were set to return a single best match to sequences in the reference DNA database.

The percentage threshold at which a sequence was identified as originating from a particular species was determined by examining how well 454 pyrosequencing sequences from control samples matched their Sanger generated sequences and by examining the amount of intra-specific and inter-specific variability within the species found in this study. We chose to construct a distance-based neighbour joining tree using Kimura-2-parameter model, as we were only interested in the ability of the 454 amplicons to delineate known species, not to examine the relationships between species. The neighbour joining tree was constructed using MEGA (available from: http://www.megasoftware.net) for COI and CytB from up to ten individuals per species for the regions sequenced in the 454 pyrosequencing experiment to examine if the shorter regions used in the 454 pyrosequencing experiment were able to accurately identify species, i.e. if each species formed a distinct group or cluster supported by high bootstraps. These sequences were extracted from the reference DNA sequence databases, as they best captured the genetic variation present in the chironomid species used in this study.

The species compositions as elucidated by the 454 pyrosequencing experiment were then compared back to the individual identifications to determine the success of 454 pyrosequencing as a tool for detecting species collected from field sites. The differences between runs (biological replicates) and genes were also examined using Spearman rank correlations to determine whether there were any differences in the way these performed. The proportion of individuals at a site was compared to the proportion of reads per individual per species from the same site, to see if relative abundances of species were reflected by the number of 454 sequences generated for each species in a sample. We also examined if there was a relationship between average number of sequences generated for both COI and CytB independently, for each species and the number of individuals for the entire 454 pyrosequencing experiment and for species that occurred at more than five field sites.

## Abbreviations

NGS: Next generation sequencing; COI: Cytochrome oxidase 1; CytB: Cytochrome b; MID: Multiplex identifier; MOTU: Molecular operational taxonomic unit; OTU: Operational taxonomic unit; RFLP: Restriction fragment length polymorphism; K2P: Kiruma-2-parameter.

## Competing interests

The authors declare that they have no competing interests.

## Authors’ contribution

MEC performed the molecular lab work, carried out the data analysis, developed the analysis pipeline, participated in the study design and drafted the manuscript. VJP and LM assisted in sourcing samples for the DNA reference database, participated in the study design and provided input into the manuscript. AAH refined the study design, performed statistical analysis and helped to draft the manuscript. All authors read and approved the final manuscript.

## Supplementary Material

Additional file 1: Table S1 GenBank accession numbers for species used in phylogenetic analysis. Includes the source and collection date of species Sanger sequenced. Click here for file

Additional file 2: Table S2 The number of sequences (reads) generated for each gene from each run (biological replicate), the total for each gene and the total for both genes for each sample. The number of individuals for each species that were present at a site is given as ‘n’. Click here for file

Additional file 3: Figure S1 PCR primer map and amplicon lengths. Primer position and PCR fragment lengths for a) COI and b) CytB used in DNA reference database construction and 454 pyrosequencing. Primer positions are indicated in blue. Click here for file

Additional file 4: Table S3 PCR fusion primers used to amplify sample for 454 pyrosequencing. Site code refers to which site each primer pair was used. Forward primers contain 454 adapter A (designated as ‘A’ at the end of the primer name) and reverse primers contain 454 adapter B (designated as ‘B’ at the end of the primer name), Each primer contains a different MID as indicated with parentheses. Click here for file
