# Supplementary material for: Environmental monitoring using next generation sequencing: rapid identification of macroinvertebrate bioindicator species
Source: Front Zool. 2013 Aug 7;10:45. doi: 10.1186/1742-9994-10-45 (PMC3750358; doi:10.1186/1742-9994-10-45)
Supplement: Additional file 1: Table S1 — GenBank accession numbers for species used in phylogenetic analysis. Includes the source and collection date of species Sanger sequenced. [file 1742-9994-10-45-S1.docx]

**Additional file 1 Table S1. GenBank accession numbers for species used in phylogenetic analysis.** Includes the source and collection date of species Sanger sequenced

| **Voucher code** | **GenBank accession no.** | | **Species** | **Source** | **Collection date** |
| --- | --- | --- | --- | --- | --- |
|  | **COI** | **CytB** |  |  |  |
| BR09Tp1 | KC750264 | KC750536 | *Ablabesmyia* sp.2 | Barwon River at Pollocksford Rd, Stonehaven, Victoria, Australia | 13-Oct-08 |
| BR08Cr3 | KC750597 | KC750266 | *Botryocladius* sp.1 | Barwon River at Pollocksford Rd, Stonehaven, Victoria, Australia | 13-Oct-08 |
| A04C384 |  | KC750712 | *Chironomus australis* | Newells Paddock Wetlands, Footscray, Victoria, Australia | 26-Apr-04 |
| S03C1129 | KC750267 | KC750604 | *Chironomus australis* | Sir William Fry Park, Moorabbin, Victoria, Australia | 28-Oct-03 |
| S03C1157 | KC750268 |  | *Chironomus australis* | Monash Gallery, Wheelers Hill, Victoria, Australia | 15-Oct-03 |
| S03C1444 | KC750269 |  | *Chironomus australis* | Box Hill Gardens, Box Hill, Victoria, Australia | 29-Oct-03 |
| S03C1469 | KC750279 | KC750719 | *Chironomus australis* | Kingston Health Reserve, Heatherton, Victoria, Australia | 28-Oct-03 |
| S03C207 | KC750270 | KC750617 | *Chironomus australis* | Shanklin upper wetland, Roxborough Park, Victoria, Australia | 22-Feb-03 |
| S03C21 | KC750271 | KC750714 | *Chironomus australis* | Spectacle Lake, Point Cook, Victoria, Australia | 23-Oct-03 |
| S03C215 | KC750272 | KC750715 | *Chironomus australis* | Station Waters, Cairnlea, Victoria, Australia | 21-Oct-03 |
| S03C336 | KC750273 |  | *Chironomus australis* | La Trobe University Moat, Bundoora, Victoria, Australia | 07-Oct-03 |
| S03C388 | KC750274 | KC750716 | *Chironomus australis* | Spavin Lake, Sunbury, Victoria, Australia | 22-Oct-03 |
| S03C389 |  | KC750590 | *Chironomus australis* | Spavin Lake, Sunbury, Victoria, Australia | 22-Oct-03 |
| S03C398 | KC750325 | KC750717 | *Chironomus australis* | Spavin Lake, Sunbury, Victoria, Australia | 22-Oct-04 |
| S03C727 | KC750278 | KC750618 | *Chironomus australis* | Lynbrook Estate Wetlands, Lynbrook Boulevard, Lynbrook Victoria, Australia | 13-Oct-03 |
| UK09C1 | KC750275 | KC750558 | *Chironomus australis* | Platypus Ponds, Sunbury, Victoria, Australia | 7-Oct-09 |
| UK09C13 | KC750276 |  | *Chironomus australis* | Platypus Ponds, Sunbury, Victoria, Australia | 7-Oct-09 |
| UK09C17 | KC750277 |  | *Chironomus australis* | Platypus Ponds, Sunbury, Victoria, Australia | 7-Oct-09 |
| A03C66 | KC750280 | KC750600 | *Chironomus cloacalis* | Red Leap, Mill Park, Victoria, Australia | 12-May-03 |
| B061 | KC750284 |  | *Chironomus cloacalis* | Ovens River, West Ovens Track, upstream of Harrietville  Victoria, Australia | 04-Jul-05 |
| B062 | KC750290 |  | *Chironomus cloacalis* | Ovens River, Snowy Creek Picnic Ground, Old Harrietville Rd,  Victoria, Australia | 04-Jul-05 |
| Cc1 | KC750289 | KF061027 | *Chironomus cloacalis* | Doncaster, Victoria, Australia | 03-Aug-01 |
| Cc2 | KC750283 |  | *Chironomus cloacalis* | Albany, Western Australia, Australia | 23-Jul-94 |
| E06C137 | KC750285 | KC750601 | *Chironomus cloacalis* | Eumemmerring Creek, 50m us railway line, Dandenong South, Victoria, Australia | 16-Nov-06 |
| E06C42 | KC750286 | KC750602 | *Chironomus cloacalis* | Dandenong Creek, Pillars Crossing, Dandenong South, Victoria, Australia | 26-Oct-06 |
| E06C85 | KC750288 | KC750605 | *Chironomus cloacalis* | Dandenong Creek, Wantirna Rd, Wantirna, Victoria, Australia | 24-Oct-06 |
| M313 |  | KC750541 | *Chironomus cloacalis* | Cherry Lake, Altona, Victoria, Australia | 09-Jan-03 |
| S03C1053 | KC750281 |  | *Chironomus cloacalis* | Nilumbik pond, Diamond Creek, Victoria, Australia | 09-Oct-03 |
| S03C1144 | KC750291 | KC750713 | *Chironomus cloacalis* | Churchill Park, Endeavour Hills, Victoria, Australia | 15-Oct-03 |
| S03C307 | KC750292 | KC750723 | *Chironomus cloacalis* | Kalparrin Reserve, Greensborough, Victoria, Australia | 24-Oct-03 |
| S03C860 | KC750287 | KC750727 | *Chironomus cloacalis* | Anakie Retarding Basin, Lara, Victoria, Australia | 10-Oct-03 |
| S03C966 | KC750282 | KC750603 | *Chironomus cloacalis* | Berwick Views, Beaconsfield, Victoria, Australia | 13-Oct-03 |
| A04C200 | KC750300 | KC750721 | *Chironomus duplex* | Albert Park Lake, Albert Park, Victoria, Australia | 26-Apr-04 |
| A04C240 | KC750301 | KC750722 | *Chironomus duplex* | Albert Park Lake, Albert Park, Victoria, Australia | 26-Apr-04 |
| A04C272 | KC750294 | KC750724 | *Chironomus duplex* | Patrick North East Wetlands, Fountain Gate, Victoria, Australia | 20-Apr-04 |
| A04C280 | KC750295 | KC750725 | *Chironomus duplex* | Patrick North East Wetlands, Fountain Gate, Victoria, Australia | 20-Apr-04 |
| A04C296 | KC750298 |  | *Chironomus duplex* | Surrey Park, Box Hill, Victoria, Australia | 27-Apr-04 |
| A04C97 | KC750302 | KC750726 | *Chironomus duplex* | Cherry Lake Wetland, Altona, Victoria, Australia | 22-Apr-04 |
| E06C44 |  | KC750728 | *Chironomus duplex* | Dandenong Creek, Pillars Crossing, Dandenong South, Victoria, Australia | 26-Oct-06 |
| LBMC5 | KC750297 | KC750729 | *Chironomus duplex* | Lake Bullen Merri, Victoria, Australia | 15-Feb-05 |
| LE09C70 | KC750293 |  | *Chironomus duplex* | Lynbrook Estate Wetlands at Lynbrook Boulevard, Lynbrook, Victoria, Australia | 6-Oct-09 |
| S03C300 | KC750299 |  | *Chironomus duplex* | Sharps Rd, Keilor, Victoria, Australia | 10-Oct-03 |
| S03C52 | KC750296 |  | *Chironomus duplex* | Spectacle Lake, Pt Cook, Victoria, Australia | 23-Oct-04 |
| S03C750 | KC750303 | KC750718 | *Chironomus duplex* | Albert Park Lake, Albert Park, Victoria, Australia | 16-Oct-03 |
| A03C138 | KC750306 | KC750609 | *Chironomus februarius* | Station Waters, Cairnlea, Victoria, Australia | 13-May-03 |
| A03C82 | KC750312 | KC750610 | *Chironomus februarius* | Shanklin upper wetland, Roxborough Park, Victoria, Australia | 12-May-03 |
| A04C275 | KC750307 | KC750611 | *Chironomus februarius* | Patrick North East Wetlands, Fountain Gate, Victoria, Australia | 20-Apr-04 |
| Cf1KF | KC750304 | KF061028 | *Chironomus februarius* | Tarampa, Queensland, Australia | 16/08/99 |
| Cf2 | KC750305 | KF061029 | *Chironomus februarius* | Hepburn, Victoria, Australia | 3/03/00 |
| S03C150 | KC750311 | KC750720 | *Chironomus februarius* | Shanklin upper wetland, Roxborough Park, Victoria, Australia | 08-Oct-03 |
| S03C216 |  | KC750615 | *Chironomus februarius* | Station Waters, Cairnlea, Victoria, Australia | 21-Oct-03 |
| S03C311 | KC750613 | KC750607 | *Chironomus februarius* | Kalparrin Reserve, Greensborough, Victoria, Australia | 24-Oct-03 |
| S03C606 | KC750614 | KC750742 | *Chironomus februarius* | Yarrunga reserve, Croydon Hills, Victoria, Australia | 14-Oct-03 |
| S03C618 | KC750310 |  | *Chironomus februarius* | Kew Billabong, Kew, Victoria, Australia | 29-Oct-03 |
| SA4C1 | KC750619 |  | *Chironomus februarius* | Waste water treatment plant, Bolivar, South Australia, Australia | 22-Jun-04 |
| SA4C6 | KC750309 |  | *Chironomus februarius* | Waste water treatment plant, Bolivar, South Australia, Australia | 22-Jun-04 |
| WA4C1 | KC750308 | KC750616 | *Chironomus februarius* | Lake Goollal, Kingsley, Western Australia, Australia | 01-Nov-04 |
| E06C291 | KC750313 | KC750743 | *Chironomus nepeanensis* | Maribyrnong River, Keilor Public Golf Course, Sydenham, Victoria, Australia | 30-Oct-06 |
| S03C1156 | KC750314 | KC750608 | *Chironomus nepeanensis* | Hill Lake, Rowville, Victoria, Australia | 15-Oct-03 |
| A04C377 | KC750315 | KC750730 | *Chironomus oppositus* | Newells Paddock Wetlands, Footscray, Victoria, Australia | 26-Apr-04 |
| A04C84 |  | KC750732 | *Chironomus oppositus* | Boar Gully, Brisbane Ranges, Victoria, Australia | 28-Apr-04 |
| E06C100 | KC750323 | KC750739 | *Chironomus oppositus* | Dandenong Creek, Jells Park, Wheelers Hill, Victoria, Australia | 14-Nov-06 |
| E06C150 | KC750735 | KC750320 | *Chironomus oppositus* | Hallam Valley Contour Drain, Centre Rd, Narre Warren South, Victoria, Australia | 16-Nov-06 |
| E06C186 | KC750318 | KC750733 | *Chironomus oppositus* | Merri Creek, Summerhill Rd, Craigieburn, Victoria, Australia | 17-Nov-06 |
| E06C320 | KC750321 | KC750736 | *Chironomus oppositus* | Maribyrnong River, Canning St Ford, Avondale Heights, Victoria, Australia | 07-Nov-06 |
| E06C4 |  | KC750738 | *Chironomus oppositus* | Dobson’s Creek, Basin-Olinda Rd opp. Wicks Reserve, The Basin, Victoria, Australia | 12-Nov-06 |
| E06C65 | KC750322 | KC750737 | *Chironomus oppositus* | Blind Creek North Branch, Forest Rd, Ferntree Gully, Victoria, Australia | 12-Nov-06 |
| E06C68 | KC750319 | KC750734 | *Chironomus oppositus* | Blind Creek North Branch, Forest Rd, Ferntree Gully, Victoria, Australia | 12-Nov-06 |
| E06C79 | KC750316 | KC750731 | *Chironomus oppositus* | Dandenong Creek, Brady Rd, Endeavour Hills, Victoria, Australia | 23-Oct-06 |
| E06C84 | KC750317 |  | *Chironomus oppositus* | Dandenong Creek, Wantirna Rd, Wantirna, Victoria, Australia | 24-Oct-06 |
| E06C67 |  | KC750591 | *Chironomus pseudoppositus* | Blind Creek North Branch, Forest Rd, Ferntree Gully, Victoria, Australia | 12-Nov-06 |
| S03C544 | KC750326 |  | *Chironomus pseudoppositus* | Brown Dam, Park Orchards, Victoria, Australia | 17-Oct-03 |
| S04C385 | KC750327 | KC750529 | *Chironomus pseudoppositus* | Don Reserve, Healesville, Victoria, Australia | 15-Oct-04 |
| S04C782 | KC750324 | KC750698 | *Chironomus pseudoppositus* | Cheltenham Rd Retarding Basin, Keysborough, Victoria, Australia | 8-OCt-04 |
| A03C775 | KC750328 | KC750697 | *Chironomus tepperi* | Alistair Knox Park, Eltham, Victoria, Australia | 27-May-03 |
| B063 | KC750329 |  | *Chironomus tepperi* | Upper Dam, Wabonga Lane, Mapley Farm  Victoria, Australia | 04-Jul-05 |
| Ctepp | KC750330 | KC750580 | *Chironomus tepperi* | Forbes, New South Wales, Australia (Lab Stock) |  |
| LE09C14 | KC750331 | KC750582 | *Chironomus tepperi* | Lynbrook Estate Wetlands at Lynbrook Boulevard, Lynbrook, Victoria, Australia | 6-Oct-09 |
| LE09C32 | KC750332 | KC750581 | *Chironomus tepperi* | Lynbrook Estate Wetlands at Lynbrook Boulevard, Lynbrook, Victoria, Australia | 6-Oct-09 |
| S03C256 | KC750333 | KC750700 | *Chironomus tepperi* | Taylors Lakes, Taylors Lakes, Victoria, Australia | 27-Oct-03 |
| S04C599 | KC750335 | KC750740 | *Chironomus tepperi* | Elsternwick Park, Brighton, Victoria, Australia | 11-Oct-04 |
| S04C676 | KC750334 | KC750699 | *Chironomus tepperi* | Woodland Park, Essendon, Victoria, Australia | 14-Oct-04 |
| S04C838 | KC750583 | KC750741 | *Chironomus tepperi* | Princess Hwy, Werribee, Victoria, Australia | 12-Oct-04 |
| S04C839 | KC750336 |  | *Chironomus tepperi* | Princess Hwy, Werribee, Victoria, Australia | 12-Oct-04 |
| S03CL15 | KC750338 |  | *Cladopelma* sp.1 | Olinda Wetlands, Lilydale, Victoria, Australia | 14-Oct-03 |
| S03CL161 | AY752680 | KC750622 | *Cladopelma* sp.1 | Mount Cooper wetlands, Bundoora, Victoria, Australia | 08-Oct-08 |
| S03CL2 | AY752675 | KC750625 | *Cladopelma* sp.1 | Don Reserve, Healesville, Victoria, Australia | 24-Oct-03 |
| S03CL33 | AY752683 |  | *Cladopelma* sp.1 | Shanklin lower wetland, Roxborough Park, Victoria, Australia | 08-Oct-03 |
| S03CL55 | AY752677 | KC750747 | *Cladopelma* sp.1 | Bulmans Rd Lake, Melton, Victoria, Australia | 21-Oct-03 |
| S03CL55 | KC750623 | KC750623 | *Cladopelma* sp.1 | Bulmans Rd Lake, Melton, Victoria, Australia | 21-Oct-03 |
| S03CL57 | AY752681 |  | *Cladopelma* sp.1 | Bulmans Rd Lake, Melton, Victoria, Australia | 21-Oct-03 |
| S03CL6 | AY752666 |  | *Cladopelma* sp.1 | Don Reserve, Healesville, Victoria, Australia | 24-Oct-03 |
| S03CL89 | KC750339 | KC750624 | *Cladopelma* sp.1 | Leed St, Doncaster East, Victoria, Australia | 17-Oct-03 |
| S04CL16 | AY752687 |  | *Cladopelma* sp.1 | Don Reserve, Healesville, Victoria, Australia | 15-Oct-04 |
| A04PN3 | KC750340 |  | *Cladopelma* sp.2 | Station Waters, Cairnlea, Victoria, Australia | 13-May-03 |
| IS04CL15 | KC750341 | KC750744 | *Cladopelma* sp.2 | Lake Goodall, Perth, Western Australia, Australia | 01-Nov-04 |
| IS04Cl16 |  | KC750745 | *Cladopelma* sp.2 | Lake Goodall, Perth, Western Australia, Australia | 01-Nov-04 |
| S03CL11 | AY752670 | KC750638 | *Cladopelma* sp.2 | Olinda Wetlands, Lilydale, Victoria, Australia | 14-Oct-03 |
| S03Cl31 | AY752676 |  | *Cladopelma* sp.2 | Red Leap, Mill Park, Victoria, Australia | 08-Oct-03 |
| S03CL53 | AY752673 | KC750586 | *Cladopelma* sp.2 | Nursery Rd, Macedon, Victoria, Australia | 22-Oct-03 |
| S03CL65 | AY752672 |  | *Cladopelma* sp.2 | Boar Gully, Brisbane Ranges, Victoria, Australia | 21-Oct-03 |
| A03T149 | KC750343 |  | *Cladotanytarsus australomancus* | Newport Lakes, Newport, Victoria, Australia | 30-May-03 |
| A03T156 | KC750344 | KC750705 | *Cladotanytarsus australomancus* | Newport Lakes, Newport, Victoria, Australia | 30-May-03 |
| A03T16 |  | DQ39386 | *Cladotanytarsus australomancus* | Olinda Wetlands, Lilydale, Victoria, Australia | 29-Apr-03 |
| A04T158 | KC750346 |  | *Cladotanytarsus australomancus* | Nilumbik pond, Diamond Creek, Victoria, Australia | 21-Apr-04 |
| E06T156 |  | KC750706 | *Cladotanytarsus australomancus* | Maribyrnong River, Arundel Road, Keilor, Victoria, Australia | 30-Oct-06 |
| E06T59 | KC750347 | KC750707 | *Cladotanytarsus australomancus* | Jacksons Creek, Sunbury Rd, Sunbury, Victoria, Australia | 03-Nov-06 |
| E06T65 | KC750348 | KC750709 | *Cladotanytarsus australomancus* | Maribyrnong River, Canning St Ford, Avondale Heights, Victoria, Australia | 07-Nov-06 |
| E06T84 | KC750350 | KC750710 | *Cladotanytarsus australomancus* | Merri Creek, Creek Pde, Westgarth, Victoria, Australia | 17-Nov-06 |
| S03T103 | DQ393841 | KC750674 | *Cladotanytarsus australomancus* | Lakeside Dr Wetland, Lara, Victoria, Australia | 10-Oct-03 |
| S03T11 | DQ393837 | KC750587 | *Cladotanytarsus australomancus* | La Trobe University Wetland, Bundoora, Victoria, Australia | 07-Oct-03 |
| S03T66 | KC750349 |  | *Cladotanytarsus australomancus* | Patrick North East Wetlands, Fountain Gate, Victoria, Australia | 6-Oct-04 |
| S03Ct17 | DQ393836 |  | *Cladotanytarsus australomancus* | Don Reserve, Healesville, Victoria, Australia | 24-Oct-03 |
| E06T118 | KC750351 |  | *Cladotanytarsus* sp.C | Maribyrnong River, McNabs Rd, Keilor, Victoria, Australia | 30-Oct-06 |
| E06T119 | KC750352 |  | *Cladotanytarsus* sp.C | Maribyrnong River, McNabs Rd, Keilor, Victoria, Australia | 30-Oct-06 |
| E06T120 | KC750345 |  | *Cladotanytarsus* sp.C | Maribyrnong River, McNabs Rd, Keilor, Victoria, Australia | 30-Oct-06 |
| E06T64 | KC750353 |  | *Cladotanytarsus* sp.C | Jacksons Creek, Homestead Way, Sunbury, Victoria, Australia | 02-Nov-06 |
| E06T80 | KC750354 |  | *Cladotanytarsus* sp.C | Jacksons Creek, Organ Pipes National Park, Keilor, Victoria, Australia | 8-Nov-06 |
| BR08 T1 |  | KF061030 | *Cladotanytarsus* sp.C | Barwon River at Pollocksford Rd, Stonehaven, Victoria, Australia | 13-Oct-08 |
| S03A6 | KC750358 | KC750595 | *Coelopynia* sp.1 | Nursery Rd, Macedon, Victoria, Australia | 22-Oct-03 |
| A04Co8 | KC750357 |  | *Coelopynia* sp.1 | Greswell Reserve, Bundoora, Victoria, Australia | 19-Apr-04 |
| A04Co9 | KC750362 |  | *Coelopynia* sp.1 | Don Reserve, Healesville, Victoria, Australia | 21-Apr-04 |
| S03Co1 | KC750359 |  | *Coelopynia* sp.1 | Liverpool RB, The Basin, Victoria, Australia | 20-Oct-03 |
| S03Co2 | KC750360 |  | *Coelopynia* sp.1 | Liverpool RB, The Basin, Victoria, Australia | 20-Oct-03 |
| S03Co3 | KC750361 |  | *Coelopynia* sp.1 | Liverpool RB, The Basin, Victoria, Australia | 20-Oct-03 |
| S04Co2 | KC750355 |  | *Coelopynia* sp.1 | Red Leap, Mill Park, Victoria, Australia | 4-Oct-04 |
| S04Co3 | KC750356 |  | *Coelopynia* sp.1 | Boardwalk, Pt Cook, Victoria, Australia | 12-Oct-04 |
| S04Cn11 | KC750363 | KC750651 | *Corynoneura scutellata* | Jacana Wetlands, Glenroy, Victoria, Australia | 5-Oct-04 |
| S04Cn12 | KC750364 | KC750652 | *Corynoneura scutellata* | Jacana Wetlands, Glenroy, Victoria, Australia | 5-Oct-04 |
| S04Cn17 | KC750365 | KC750556 | *Corynoneura scutellata* | Cala St Ponds, West Footscray, Victoria, Australia | 14-Oct-04 |
| S04Cn3 | KC750366 | KC750653 | *Corynoneura scutellata* | Monbulk retarding basin, Belgrave, Victoria, Australia | 6-Oct-04 |
| S04Cn4 |  | KC750557 | *Corynoneura scutellata* | Princess Hwy and Ring Rd Interchange, Laverton, Victoria, Australia | 12-Oct-04 |
| SK09Cr2 | KC750367 | KC750596 | *Corynoneura scutellata* | Shankland Wetland, Meadow Heights | 12-Oct-09 |
| DKI4 | KC750372 | KC750573 | *Cricotopus albitarsis* | Dandenong Creek, Kidds Rd, Dandenong, Victoria, Australia | 26-Oct-06 |
| JSS6 | KC750373 | KC750574 | *Cricotopus albitarsis* | Jacksons Creek, Sunbury Rd, Sunbury, Victoria, Australia | 3-Nov-06 |
| MBF7 | KC750370 | KC750571 | *Cricotopus albitarsis* | Maribyrnong River, Brimbank Park Ford upstream Taylors Creek & Keilor TP, Victoria, Australia | 1-Nov-06 |
| A04Cr4 | KC750369 | KC750570 | *Cricotopus albitarsis* | Kalparrin Reserve, Greensborough, Victoria, Australia | 19-Apr-04 |
| A04Cr5 | KC750371 | KC750572 | *Cricotopus albitarsis* | La Trobe University Wetland, Bundoora, Victoria, Australia | 14-May-03 |
| CrM20 | KC750569 |  | *Cricotopus albitarsis* | Maribyrnong River Jacksons riffle, Keilor, Victoria, Australia | 23-Jun-02 |
| E06Cr191 | KC750375 |  | *Cricotopus albitarsis* | Maribyrnong River, Brimbank Park Ford upstream Taylors Creek & Keilor TP, Victoria, Australia | 1-Nov-06 |
| E06Cr204 | KC750392 | KC750685 | *Cricotopus albitarsis* | Kororoit Creek, Warmington Rd, Sunshine, Victoria, Australia | 10-Nov-06 |
| E06Cr233 | KC750374 |  | *Cricotopus albitarsis* | Kororoit Creek, Princes Hwy, Brooklyn, Victoria, Australia | 10-Nov-06 |
| M201 | KC750379 |  | *Cricotopus albitarsis* | Darebin Creek Riffle, Reservoir, Victoria, Australia | 20-Dec-02 |
| CN3 | KC750378 |  | *Cricotopus annuliventris* | Campaspe River, Redesdale, Victoria, Australia | 2012 |
| E06Cr88 | KC750377 |  | *Cricotopus annuliventris* | Dandenong Creek, Dobson’s Lane bridge, Victoria, Australia | 24-Oct-06 |
| ME09Cr3 | KC750376 |  | *Cricotopus annuliventris* | Brodies Lakes at Greenvale Reservoir Park, Greenvale, Victoria, Australia | 12-Oct-09 |
| S04Cr8 | KC750337 | KC750753 | *Cricotopus annuliventris* | Bolin Bolin Billabong, Bulleen, Victoria, Australia | 13-Oct-04 |
| E06Cr12 | KC750380 | KC750676 | *Cricotopus* sp.1 | Kororoit Creek, Warmington Rd, Sunshine, Victoria, Australia | 10-Nov-06 |
| E06Cr26 | KC750381 | KC750678 | *Cricotopus* sp.1 | Dandenong Creek, Wellington Rd, Rowville, Victoria, Australia | 12-Nov-06 |
| E06Cr272 | KC750383 | KC750679 | *Cricotopus* sp.1 | Merri Creek, Barry Rd, Campbellfield, Victoria, Australia | 17-Nov-06 |
| E06Cr91 |  | KC750680 | *Cricotopus* sp.1 | Dandenong Creek, Dobson’s Lane bridge, Victoria, Australia | 24-Oct-06 |
| JSS1 | KC750389 |  | *Cricotopus* sp.1 | Jacksons Creek, Sunbury Rd, Sunbury, Victoria, Australia | 3-Nov-06 |
| S03Cr1 |  | KC750677 | *Cricotopus* sp.1 | Olinda Wetlands, Lilydale, Victoria, Australia | 14-Oct-03 |
| S03Cr108 | KC750386 |  | *Cricotopus* sp.1 | Spectacle Lake, Pt Cook, Victoria, Australia | 23-Oct-04 |
| S03Cr146 | KC750388 |  | *Cricotopus* sp.1 | Yarra Glen Wetlands, Yarra Glen, Victoria, Australia | 24-Oct-03 |
| S03Cr164 | KC750382 |  | *Cricotopus* sp.1 | Goldentree Wetlands, Chirnside Park, Victoria, Australia | 14-Oct-03 |
| S03Cr91 | KC750384 | KC750761 | *Cricotopus* sp.1 | Monbolk Retarding Basin, Belgrave, Victoria, Australia | 20-Oct-03 |
| S04Cr10 | KC750387 |  | *Cricotopus* sp.1 | Jack Holt Way, Epsom Estate, Victoria, Australia | 7-Oct-04 |
| S04Cr24 | KC750385 | KC750683 | *Cricotopus* sp.1 | Ringwood Lake, Ringwood, Victoria, Australia | 13-Oct-04 |
| BR08Cr1 | KC750390 | KC750578 | *Cricotopus* sp.1 | Barwon River at Pollocksford Rd, Stonehaven, Victoria, Australia | 13-Oct-08 |
| CrM1 | KC750687 |  | *Cricotopus* sp.2 | Maribyrnong River Jacksons riffle, Keilor, Victoria, Australia | 23-Jun-02 |
| CrM20 | KC750368 | KC750394 | *Cricotopus* sp.2 | Maribyrnong River Jacksons riffle, Keilor, Victoria, Australia | 23-Jun-02 |
| DKI2 | KC750395 |  | *Cricotopus* sp.2 | Dandenong Creek, Kidds Rd, Dandenong, Victoria, Australia | 26-Oct-06 |
| E06Cr128 |  | KC750688 | *Cricotopus* sp.2 | Eumemmerring Creek, 50m us railway line, Dandenong South, Victoria, Australia | 16-Nov-06 |
| E06Cr164 | KC750396 |  | *Cricotopus* sp.2 | Maribyrnong River, Calder Highway, Keilor, Victoria, Australia | 1-Nov-06 |
| JSS8 | KC750398 | KC750576 | *Cricotopus* sp.2 | Jacksons Creek, Sunbury Rd, Sunbury, Victoria, Australia | 3-Nov-06 |
| M186 |  | KC750689 | *Cricotopus* sp.2 | Darebin Creek Riffle, Reservoir, Victoria, Australia | 20-Dec-02 |
| M203 |  | KC750690 | *Cricotopus* sp.2 | Darebin Creek Riffle, Reservoir, Victoria, Australia | 20-Dec-02 |
| M42 | KC750391 | KC750691 | *Cricotopus* sp.2 | Woori Yallock Creek riffle, Woori Yallock, Victoria, Australia | 23-Aug-02 |
| MCA11 | KC750397 |  | *Cricotopus* sp.2 | Maribyrnong River, McNabs Road, Keilor, Victoria, Australia | 30-Oct-06 |
| MCA5 |  | KC750577 | *Cricotopus* sp.2 | Maribyrnong River, McNabs Road, Keilor, Victoria, Australia | 30-Oct-06 |
| S03Cr170 | KC750393 | KC750575 | *Cricotopus* sp.2 | Jacana Wetlands, Glenroy, Victoria, Australia | 27-Oct-03 |
| A03D60 | KC750399 | KC750549 | *Dicrotendipes pseudoconjunctus* | La Trobe University Wetland, Bundoora, Victoria, Australia | 14-May-03 |
| A03T153 | KC750403 | KC750554 | *Dicrotendipes pseudoconjunctus* | Newport Lakes, Newport, Victoria, Australia | 30-May-03 |
| E06D65 |  | KC750550 | *Dicrotendipes pseudoconjunctus* | Kororoit Creek, Warmington Rd, Sunshine, Victoria, Australia | 10-Nov-06 |
| LE09C51 | KC750400 | KC750551 | *Dicrotendipes pseudoconjunctus* | Lynbrook Estate Wetlands at Lynbrook Boulevard, Lynbrook, Victoria, Australia | 6-Oct-09 |
| LE09D7 | KC750404 | KC750555 | *Dicrotendipes pseudoconjunctus* | Lynbrook Estate Wetlands at Lynbrook Boulevard, Lynbrook, Victoria, Australia | 6-Oct-09 |
| MC09C2 | KC750401 | KC750552 | *Dicrotendipes pseudoconjunctus* | Maribyrnong River at Caulder Hwy, Keilor, Victoria, Australia | 7-Oct-09 |
| S03D10 | KC750406 | KC750708 | *Dicrotendipes pseudoconjunctus* | Spectacle Lake, Pt Cook, Victoria, Australia | 23-Oct-04 |
| S03D26 | KC750408 |  | *Dicrotendipes pseudoconjunctus* | Shanklin upper wetland, Roxborough Park, Victoria, Australia | 08-Oct-03 |
| S03D280 | KC750407 |  | *Dicrotendipes pseudoconjunctus* | Hartley Rd Reserve, Wonga Park, Victoria, Australia | 29-Oct-03 |
| S03D34 | KC750405 | KC750702 | *Dicrotendipes pseudoconjunctus* | Station Waters, Cairnlea, Victoria, Australia | 21-Oct-03 |
| S03D83 |  | KC750636 | *Dicrotendipes pseudoconjunctus* | Boar Gully, Brisbane Ranges, Victoria, Australia | 21-Oct-03 |
| S03D94 | KC750421 | KC750692 | *Dicrotendipes pseudoconjunctus* | Jack Holt Way, Epsom Estate, Victoria, Australia | 28-Oct-03 |
| S03D96 | KC750402 |  | *Dicrotendipes pseudoconjunctus* | Berwick Springs Lake, Berwick Springs, Victoria, Australia | 13-Oct-03 |
| A04D60 |  | KC750703 | *Dicrotendipes septemmaculatus* | Albert Park Lake, Albert Park, Victoria, Australia | 26-Apr-04 |
| E06D11 | KC750409 |  | *Dicrotendipes septemmaculatus* | Maribyrnong River, Canning St Ford, Avondale Heights Victoria, Australia | 7-Nov-06 |
| E06D38 | KC750410 |  | *Dicrotendipes septemmaculatus* | Kororoit Creek, Main Rd West, St Albans, Victoria, Australia | 09-Nov-06 |
| MM09D1 | KC750411 |  | *Dicrotendipes septemmaculatus* | Highlands Wetland Estate, Cragieburn, Victoria, Australia | 9-Oct-09 |
| S03CL48 | KC750412 | KC750641 | *Dicrotendipes septemmaculatus* | La Trobe University Wetland, Bundoora, Victoria, Australia | 07-Oct-03 |
| S03D281 | KC750413 |  | *Dicrotendipes septemmaculatus* | Hartley Rd Reserve, Wonga Park, Victoria, Australia | 29-Oct-03 |
| S03D68 | KC750414 | KC750585 | *Dicrotendipes septemmaculatus* | La Trobe University Wetland, Bundoora, Victoria, Australia | 07-Oct-03 |
| A04D31 | KC750417 |  | *Dicrotendipes* sp.4 | Boar Gully, Brisbane Ranges, Victoria, Australia | 28-Apr-04 |
| A04T133 | KC750415 |  | *Dicrotendipes* sp.4 | Berwick Views, Beaconsfield, Victoria, Australia | 20-Apr-04 |
| S03D291 | KC750416 |  | *Dicrotendipes* sp.4 | Woodland Park, Essendon, Victoria, Australia | 30-Oct-03 |
| S03D56 | KC750418 | KC750635 | *Dicrotendipes* sp.4 | La Trobe University Moat, Bundoora, Victoria, Australia | 07-Oct-03 |
| S03D76 | KC750419 | KC750584 | *Dicrotendipes* sp.4 | Liverpool retarding basin, The Basin, Victoria, Australia | 20-Oct-03 |
| S04D136 | KC750420 | KC750701 | *Dicrotendipes* sp.4 | Ringwood Lake, Ringwood, Victoria, Australia | 13-Oct-04 |
| A03D112 | KC750430 |  | *Dicrotendipes* sp.A | Blackburn Lake Wetlands, Blackburn, Victoria, Australia | 29-May-03 |
| A03D115 | KC750429 | KC750548 | *Dicrotendipes* sp.A | Blackburn Lake Wetlands, Blackburn, Victoria, Australia | 29-May-03 |
| E06D1 | KC750428 | KC750632 | *Dicrotendipes* sp.A | Blind Creek, Scoresby Rd, Wantirna South, Victoria, Australia | 12-Nov-06 |
| E06D5 | KC750634 | KC750427 | *Dicrotendipes* sp.A | Blind Creek, Scoresby Rd, Wantirna South, Victoria, Australia | 12-Nov-06 |
| GC09K3 | KC750426 | KC750547 | *Dicrotendipes* sp.A | Gardiners Creek at High St, Glen Iris, Victoria, Australia | 13-Oct-09 |
| M284 | KC750425 | KC750631 | *Dicrotendipes* sp.A | Storm water outfall Blackburn Lake, Blackburn, Victoria, Australia | 03-Jan-03 |
| M309 | KC750424 | KC750633 | *Dicrotendipes* sp.A | Storm water outfall Blackburn Lake, Blackburn, Victoria, Australia | 03-Jan-03 |
| M373 | KC750423 |  | *Dicrotendipes* sp.A | Wetland, Heatherton Rd, Victoria, Australia | 09-Jan-00 |
| S03Cr148 | KC750422 | KC750684 | *Dicrotendipes* sp.A | Blackburn Lake Wetlands, Blackburn, Victoria, Australia | 17-Oct-03 |
| A03K31 | KC750434 | KC750643 | *Dicrotendipes* sp.A | Don Reserve, Healesville, Victoria, Australia | 28-Apr-03 |
| E06K2 | KC750432 | KC750646 | *Kiefferulus cornishi* | Kororoit Creek, Sinclair’s Rd, Melton East, Victoria, Australia | 9-Nov-06 |
| E06K25 | KC750433 | KC750644 | *Kiefferulus cornishi* | Merri Creek, Summerhill Rd, Craigieburn, Victoria, Australia | 17-Nov-06 |
| IS04K6 | KC750435 |  | *Kiefferulus cornishi* | Homebush, Sydney, New South Wales, Australia | 01-Apr-03 |
| S03K64 | KC750436 |  | *Kiefferulus cornishi* | Hedgely Dene, Caulfield, Victoria, Australia | 16-Oct-03 |
| A03K106 | KC750437 |  | *Kiefferulus intertinctus* | Kew Billabong, Kew, Victoria, Australia | 19-May-03 |
| A03K11 | KC750431 | KC750642 | *Kiefferulus intertinctus* | Kew Billabong, Kew, Victoria, Australia | 19-May-03 |
| S03K1 | KC750438 | KC750553 | *Kiefferulus intertinctus* | Shanklin upper wetland, Roxborough Park, Victoria, Australia | 08-Oct-03 |
| S03K10 | KC750439 | KC750647 | *Kiefferulus intertinctus* | Sharps Rd, Keilor, Victoria, Australia | 10-Oct-03 |
| S03K19 | KC750440 | KC750648 | *Kiefferulus intertinctus* | Kalparrin Reserve, Greensborough, Victoria, Australia | 24-Oct-03 |
| S03K30 | KC750441 |  | *Kiefferulus intertinctus* | La Trobe University Moat, Bundoora, Victoria, Australia | 07-Oct-03 |
| S03K48 | KC750443 | KC750770 | *Kiefferulus intertinctus* | Cherry Lake Wetland, Altona, Victoria, Australia | 23-Oct-03 |
| S03K53 | KC750444 |  | *Kiefferulus intertinctus* | Yarrunga Reserve, Croydon Hills, Victoria, Australia | 14-Oct-03 |
| S03K56 | KC750442 | KC750650 | *Kiefferulus intertinctus* | Kew Billabong, Kew, Victoria, Australia | 29-Oct-03 |
| S03K63 | KC750445 | KC750649 | *Kiefferulus intertinctus* | Hedgely Dene, Caulfield, Victoria, Australia | 16-Oct-03 |
| S03K65 | KC750446 | KC750645 | *Kiefferulus intertinctus* | Berwick Views, Beaconsfield, Victoria, Australia | 13-Oct-03 |
| KM4 | KC750449 |  | *Kiefferulus martini* | Glynn's Wetland, Warrandyte, Victoria, Australia | 2006 |
| S03D84 | AY752665 |  | *Kiefferulus martini* | Boar Gully, Brisbane Ranges, Victoria, Australia | 21-Oct-03 |
| S04Km1 | KC750451 | KC750694 | *Kiefferulus martini* | La Trobe University Wetland, Bundoora, Victoria, Australia | 15-Oct-04 |
| S04Km2 | KC750447 | KC750696 | *Kiefferulus martini* | Bolin Bolin Billabong, Bulleen, Victoria, Australia | 13-Oct-04 |
| S04Km3 | KC750450 | KC750693 | *Kiefferulus martini* | Red Leap, Mill Park, Victoria, Australia | 4-Oct-04 |
| S04Km4 | KC750452 | KC750695 | *Kiefferulus martini* | Don Reserve, Healesville, Victoria, Australia | 15-Oct-04 |
| IS04CL10 | KC750453 |  | *Microchironomus forcipatus* | Torren's River, South Australia, Australia | 01-Apr-04 |
| IS04CL4 | KC750454 | KC750637 | *Microchironomus forcipatus* | Torren's River, South Australia, Australia | 01-Apr-04 |
| S03CL102 | AY752685 | KC750629 | *Microchironomus forcipatus* | Healthy Bay Wetlands, Dandenong North, Victoria, Australia | 15-Oct-03 |
| S03CL116 | KC750640 | KC750746 | *Microchironomus forcipatus* | Berwick Views, Beaconsfield, Victoria, Australia | 13-Oct-03 |
| S03CL134 | AY752664 | KC750620 | *Microchironomus forcipatus* | Monbolk retarding basin, Belgrave, Victoria, Australia | 20-Oct-03 |
| S03CL26 | AY752668 | KC750639 | *Microchironomus forcipatus* | Red Leap, Mill Park, Victoria, Australia | 8-Oct-03 |
| S03CL28 | KC750455 | KC750704 | *Microchironomus forcipatus* | Red Leap, Mill Park, Victoria, Australia | 8-Oct-03 |
| S03CL34 | AY752674 | KC750621 | *Microchironomus forcipatus* | Shanklin lower wetland, Roxborough Park, Victoria, Australia | 08-Oct-03 |
| S04CL169 | KC750456 |  | *Microchironomus forcipatus* | Surrey Park, Box Hill, Victoria, Australia | 6-Oct-04 |
| S03Ch4 | KC750457 |  | *Parachironomus delinificus* | Lagoon Park, Caulder Hwy Macedon, Victoria, Australia | 22-Oct-03 |
| S03Pa13 | KC750458 |  | *Parachironomus delinificus* | Glynn's Wetland, Warrandyte, Victoria, Australia | 24-Oct-03 |
| S03Pa8 | KC750459 |  | *Parachironomus delinificus* | Bolin Bolin Billabong, Bulleen, Victoria, Australia | 29-Oct-03 |
| S04D96 | KC750460 | KC750628 | *Parachironomus delinificus* | Basterfield Park, Moorabbin, Victoria, Australia | 8-Oct-04 |
| S04Pa13 |  | KC750626 | *Parachironomus delinificus* | Glynn's Wetland, Warrandyte, Victoria, Australia | 15-Oct-04 |
| S04Pa14 |  | KC750544 | *Parachironomus delinificus* | Glynn's Wetland, Warrandyte, Victoria, Australia | 15-Oct-04 |
| BR08C4 | KC750461 |  | *Parachironomus* sp.3 | Barwon River at Pollocksford Rd, Stonehaven, Victoria, Australia | 13-Oct-08 |
| BR08R5 | KC750462 | KC750542 | *Parachironomus* sp.3 | Barwon River at Pollocksford Rd, Stonehaven, Victoria, Australia | 13-Oct-08 |
| BR08R6 | KC750463 | KC750543 | *Parachironomus* sp.3 | Barwon River at Pollocksford Rd, Stonehaven, Victoria, Australia | 13-Oct-08 |
| E06Cr124 |  | KC750765 | *Paralimnophyes* sp.1 | Eumemmerring Creek, Belgrave-Hallam Rd, Lysterfield, Victoria, Australia | 14-Nov-06 |
| E06Cr150 | KC750767 |  | *Paralimnophyes* sp.1 | Dunlop’s Drain, Junction Rd, Dingley Village, Victoria, Australia | 14-Nov-06 |
| E06Cr39 | KC750766 |  | *Paralimnophyes* sp.1 | Dandenong Creek, Pillars Crossing, Dandenong South, Victoria, Australia | 26-Oct-06 |
| ME09Cr1 | KC750464 | KC750599 | *Paralimnophyes* sp.1 | Brodies Lakes at Greenvale Reservoir Park, Greenvale, Victoria, Australia | 12-Oct-09 |
| ME09Cr2 | KC750465 | KC750598 | *Paralimnophyes* sp.1 | Brodies Lakes at Greenvale Reservoir Park, Greenvale, Victoria, Australia | 12-Oct-09 |
| ME09Cr5 | KC750466 |  | *Paralimnophyes* sp.1 | Brodies Lakes at Greenvale Reservoir Park, Greenvale, Victoria, Australia | 12-Oct-09 |
| DB09Tp1 | KC750263 |  | *Paramerina* sp.4 | Deep Creek at Bulla Rd, Bulla, Victoria, Australia | 7-Oct-09 |
| DB09Tp2 |  | KC750537 | *Paramerina* sp.4 | Deep Creek at Bulla Rd, Bulla, Victoria, Australia | 7-Oct-09 |
| E06Tp10 | KC750535 | KC750540 | *Paramerina* sp.4 | Blind Creek, Scoresby Rd, Wantirna South, Victoria, Australia | 12-Nov-06 |
| E06Tp11 |  | KC750539 | *Paramerina* sp.4 | Maribyrnong River, Canning St Ford, Avondale Heights Victoria, Australia | 7-Nov-06 |
| E06Tp8 |  | KC750538 | *Paramerina* sp.4 | Maribyrnong River, Calder Highway, Keilor, Victoria, Australia | 1-Nov-06 |
| E06T69 |  | KC750589 | *Paramerina* sp.4 | Dandenong Creek, Doongala Forest Access Rd, Dandenong Ranges National park, Victoria, Australia | 19-Oct-06 |
| A03T143 | JN855597 | JN855641 | *Paratanytarsus grimmii* | Boardwalk, Pt Cook, Victoria, Australia | 30-May-03 |
| A04T38 | JN855642 | JN855598 | *Paratanytarsus grimmii* | Navan Park, Melton, Victoria, Australia | 28-Apr-04 |
| C10p1 | JN855600 | JN855644 | *Paratanytarsus grimmii* | St Lawrence River, Quebec Canada | 15-Aug-06 |
| DBO3.5 | JN855639 | JN855631 | *Paratanytarsus grimmii* | Glynn's Wetland, Warrandyte, Victoria, Australia | 1-Jan-07 |
| DOL1.2 | JN855647 | JN855603 | *Paratanytarsus grimmii* | Dandenong Creek, Dobson’s Lane bridge, Victoria, Australia | 24-Oct-06 |
| DWA3.1 | JN855649 | JN855605 | *Paratanytarsus grimmii* | Dandenong Creek, Wantirna Rd, Wantirna, Victoria, Australia | 24-Oct-06 |
| E06T20 |  | KC750711 | *Paratanytarsus grimmii* | Eumemmerring Creek, Belgrave-Hallam Rd, Lysterfield, Victoria, Australia | 14-Nov-06 |
| E06T9 | KC750630 |  | *Paratanytarsus grimmii* | Dandenong Creek, Kidds Rd, Dandenong, Victoria, Australia | 26-Oct-06 |
| GermC11 | JN855665 | JN855621 | *Paratanytarsus grimmii* | Berlin, Germany | 01-Aug-09 |
| J12 | JN855657 | JN855613 | *Paratanytarsus grimmii* | Yamazaki River, Nagoya, Japan | laboratory culture collected in 1992 |
| JEB4.2 | JN855653 | JN855609 | *Paratanytarsus grimmii* | Glynn's Wetland, Warrandyte, Victoria, Australia | 09-Jan-07 |
| UKAg12 | JN855662 | JN855618 | *Paratanytarsus grimmii* | Ardleigh Reservoir, England | 15-Sep-09 |
| E06T95 | KC750545 | KC750467 | *Paratanytarsus sp.D* | Merri Creek, Summerhill Rd, Craigieburn, Victoria, Australia | 17-Nov-06 |
| E06T98 | KC750468 | KC750546 | *Paratanytarsus* sp.D | Merri Creek, Summerhill Rd, Craigieburn, Victoria, Australia | 17-Nov-06 |
| E06T99 | KC750469 |  | *Paratanytarsus* sp.D | Merri Creek, Summerhill Rd, Craigieburn, Victoria, Australia | 17-Nov-06 |
| ME09T1 | KC750470 |  | *Paratanytarsus* sp.D | Brodies Lakes at Greenvale Reservoir Park, Greenvale, Victoria, Australia | 12-Oct-09 |
| A03Cr10 |  | KC750749 | *Paratrichocladius* sp.1 | Yarra Glen Wetland, Yarra Glen, Victoria, Australia | 29-May-03 |
| A04Cr10 | KC750471 | KC750750 | *Paratrichocladius* sp.1 | Lynbrook Estate Wetlands, Lynbrook Estate, Victoria, Australia | 07-Apr-04 |
| A04Cr15 |  | KC750755 | *Paratrichocladius* sp.1 | Lynbrook Estate Wetlands, Lynbrook Estate, Victoria, Australia | 07-Apr-04 |
| A04Cr20 |  | KC750756 | *Paratrichocladius* sp.1 | Lynbrook Estate Wetlands, Lynbrook Estate, Victoria, Australia | 07-Apr-04 |
| A04Cr39 | KC750472 | KC750754 | *Paratrichocladius* sp.1 | Crawley Rd Dam, Narre Warren, Victoria, Australia | 20-Apr-04 |
| A04Cr56 | KC750475 | KC750751 | *Paratrichocladius* sp.1 | Yarra Glen Wetlands, Yarra Glen, Victoria, Australia | 29-May-03 |
| E06T60 |  | KC750760 | *Paratrichocladius* sp.1 | Jacksons Creek, Sunbury Rd, Sunbury, Victoria, Australia | 3-Nov-06 |
| M48 | KC750757 |  | *Paratrichocladius* sp.1 | King Parrot Creek, Kinglake, Victoria, Australia | 26-Dec-02 |
| MM09Cr1 | KC750488 | KC750594 | *Paratrichocladius* sp.1 | Highlands Wetland Estate, Craigieburn, Victoria, Australia | 9-Oct-09 |
| S04Cr54 | KC750473 | KC750763 | *Paratrichocladius* sp.1 | Jacana Wetlands, Glenroy, Victoria, Australia | 5-Oct-04 |
| S04Cr61 | KC750474 | KC750758 | *Paratrichocladius* sp.1 | Jacana Wetlands, Glenroy, Victoria, Australia | 5-Oct-04 |
| S04Cr66 | KC750476 | KC750764 | *Paratrichocladius* sp.1 | Terror Street Wetlands, Keilor, Victoria, Australia | 14-Oct-04 |
| S04Cr67 | KC750477 | KC750759 | *Paratrichocladius* sp.1 | Fitzgerald Rd Wetland, Sunshine, Victoria, Australia | 14-Oct-04 |
| E06Cr147 | KC750479 |  | *Paratrichocladius* sp.2 | Eumemmerring Creek, 50m us railway line, Dandenong South, Victoria, Australia | 16-Nov-06 |
| E06Cr275 | KC750478 |  | *Paratrichocladius* sp.2 | Merri Creek, O'Herns Rd, Somerton, Victoria, Australia | 17-Nov-06 |
| Pt8 | KC750480 |  | *Paratrichocladius* sp.2 | Melbourne,, Victoria, Australia, Victoria, Australia |  |
| S03Cr128 | KC750481 | KC750682 | *Paratrichocladius* sp.2 | Nilumbik pond, Diamond Creek, Victoria, Australia | 09-Oct-03 |
| S03Cr64 | KC750482 | KC750686 | *Paratrichocladius* sp.2 | Lynbrook Estate Wetlands, Lynbrook Estate, Victoria, Australia | 13-Oct-03 |
| S04Cr30 | KC750762 | KC750681 | *Paratrichocladius* sp.2 | Boardwalk, Point Cook, Victoria, Australia | 12-Oct-04 |
| S04Cr33 | KC750483 |  | *Paratrichocladius* sp.2 | Queens Park, Moonee Ponds, Victoria, Australia | 14-Oct-04 |
| S04Cr65 | KC750485 | KC750752 | *Paratrichocladius* sp.2 | Princess Hwy and Ring Rd Interchange, Laverton, Victoria, Australia | 12-Oct-04 |
| SK09Cr1 | KC750484 | HQ248020 | *Paratrichocladius* sp.2 | Shankland Wetland, Meadow Heights | 12-Oct-09 |
| BR08R2 | KC750486 | KC750592 | *Polypedilum convexum* | Barwon River at Pollocksford Rd, Stonehaven, Victoria, Australia | 13-Oct-08 |
| BR08R3 | KC750487 | KC750593 | *Polypedilum convexum* | Barwon River at Pollocksford Rd, Stonehaven, Victoria, Australia | 13-Oct-08 |
| A04PN10 | KC750489 | KC750768 | *Polypedilum nubifer* | Monash Gallery, Wheelers Hill, Victoria, Australia | 20-Apr-04 |
| A04PN12 | KC750490 | KC750775 | *Polypedilum nubifer* | Ruffey Lake, Doncaster, Victoria, Australia | 27-Apr-04 |
| A04PN4 | KC750493 | KC750774 | *Polypedilum nubifer* | Middle Rd Pond, Caroline Springs, Victoria, Australia | 28-Apr-04 |
| A04PN5 | KC750491 |  | *Polypedilum nubifer* | Greswell Reserve, Bundoora, Victoria, Australia | 19-Apr-04 |
| A04PN6 | KC750492 | KC750772 | *Polypedilum nubifer* | Cherry Lake Wetland, Altona, Victoria, Australia | 22-Apr-04 |
| A04PN7 | KC750494 | KC750769 | *Polypedilum nubifer* | Lynbrook Estate Wetlands, Lynbrook Estate, Victoria, Australia | 07-Apr-04 |
| A04PN9 | KC750495 |  | *Polypedilum nubifer* | Nilumbik Pond, Diamond Creek, Victoria, Australia | 21-Apr-04 |
| S03K4 | AY752679 |  | *Polypedilum nubifer* | Shanklin upper wetland, Roxborough Park, Victoria, Australia | 08-Oct-03 |
| S03Po39 | KC750509 | KC750627 | *Polypedilum nubifer* | Olinda Wetlands, Lilydale, Victoria, Australia | 14-Oct-03 |
| S03Po4 | KC750496 |  | *Polypedilum nubifer* | Olinda Wetlands, Lilydale, Victoria, Australia | 14-Oct-03 |
| SA4PN2 | KC750497 | KC750612 | *Polypedilum nubifer* | Waste water treatment plant, Bolivar, South Australia, Australia | 22-Jun-04 |
| E06Po101 | KC750498 | KC750559 | *Polypedilum* sp.C | Maribyrnong River, Canning St Ford, Avondale Heights Victoria, Australia | 07-Nov-06 |
| E06Po102 | KC750511 |  | *Polypedilum* sp.C | Maribyrnong River, Canning St Ford, Avondale Heights Victoria, Australia | 07-Nov-06 |
| E06Po103 |  | KC750655 | *Polypedilum* sp.C | Maribyrnong River, Canning St Ford, Avondale Heights Victoria, Australia | 07-Nov-06 |
| E06Po107 | KC750512 | KC750658 | *Polypedilum* sp.C | Maribyrnong River, Keilor Public Golf Course, Sydenham, Victoria, Australia | 30-Oct-06 |
| E06Po118 | KC750513 |  | *Polypedilum* sp.C | Maribyrnong River, Brimbank Park Ford upstream Taylors Creek & Keilor TP, Victoria, Australia | 01-Nov-06 |
| E06Po119 | KC750499 | KC750663 | *Polypedilum* sp.C | Maribyrnong River, Brimbank Park Ford upstream Taylors Creek & Keilor TP, Victoria, Australia | 01-Nov-06 |
| E06Po128 | KC750514 | KC750654 | *Polypedilum* sp.C | Maribyrnong River, Canning St Ford, Avondale Heights Victoria, Australia | 07-Nov-06 |
| E06Po132 | KC750500 | KC750659 | *Polypedilum* sp.C | Jacksons Creek, Organ Pipes National Park, Keilor, Victoria, Australia | 8-Nov-06 |
| E06Po25 |  | KC750661 | *Polypedilum* sp.C | Dandenong Creek, Jells Park, Wheelers Hill, Victoria, Australia | 39035 |
| E06Po40 | KC750516 | KC750656 | *Polypedilum* sp.C | Merri Creek, Summerhill Rd, Craigieburn, Victoria, Australia | 17-Nov-06 |
| E06Po50 | KC750515 | KC750657 | *Polypedilum* sp.C | Merri Creek, Summerhill Rd, Craigieburn, Victoria, Australia | 17-Nov-06 |
| E06Po75 | KC750517 | KC750660 | *Polypedilum* sp.C | Jacksons Creek, Homestead Way, Sunbury, Victoria, Australia | 2-Nov-06 |
| E06Po84 | KC750662 | KC750501 | *Polypedilum* sp.C | Maribyrnong River, Calder Highway, Keilor, Victoria, Australia | 1-Nov-06 |
| MC09C3 | KC750502 | KC750560 | *Polypedilum* sp.C | Maribyrnong River at Caulder Hwy, Keilor, Victoria, Australia | 7-Oct-09 |
| E06Po126 | KC750504 |  | *Polypedilum* sp.E | Maribyrnong River, Canning St Ford, Avondale Heights Victoria, Australia | 07-Nov-06 |
| E06Po85 | KC750503 |  | *Polypedilum* sp.E | Maribyrnong River, Calder Highway, Keilor, Victoria, Australia | 1-Nov-06 |
| E06Po86 | KC750505 | KC750561 | *Polypedilum* sp.E | Maribyrnong River, Calder Highway, Keilor, Victoria, Australia | 1-Nov-06 |
| E06Po87 | KC750506 | KC750664 | *Polypedilum* sp.E | Maribyrnong River, Calder Highway, Keilor, Victoria, Australia | 1-Nov-06 |
| E06Po92 | KC750507 | KC750562 | *Polypedilum* sp.E | Maribyrnong River, Calder Highway, Keilor, Victoria, Australia | 1-Nov-06 |
| E06Po94 | KC750508 |  | *Polypedilum* sp.E | Maribyrnong River, Calder Highway, Keilor, Victoria, Australia | 01-Nov-06 |
| E06Po96 | KC750510 |  | *Polypedilum* sp.E | Maribyrnong River, Calder Highway, Keilor, Victoria, Australia | 01-Nov-06 |
| M259 |  | KC750665 | *Polypedilum* sp.E | Yarra River, Fitzsimmons Lane, Eltham, Victoria, Australia | 03-Jan-03 |
| Pr124 | HQ248061 | HQ248016 | *Procladius paludicola* | Boar Gully, Brisbane ranges, Victoria, Australia | 27-Feb-03 |
| Pr239 | HQ248063 | HQ248012 | *Procladius paludicola* | Skeleton Creek, Sayers Rd, Victoria, Australia | 07-Jan-02 |
| Pr335 | HQ248064 | HQ248021 | *Procladius paludicola* | Lake Belvedere, Sydney, New South Wales, Australia, Victoria, Australia | 01-Apr-03 |
| Pr348 | HQ248049 | HQ248019 | *Procladius paludicola* | Corridor Wetlands, Sydney, New South Wales, Australia, Victoria, Australia | 01-Apr-03 |
| Pr48 | HQ248062 | HQ248015 | *Procladius paludicola* | Monbulk Retarding Basin, Belgrave, Victoria, Australia | 24-Feb-03 |
| SAPr7 | HQ248057 | HQ248024 | *Procladius paludicola* | Waste water treatment plant, Bolivar, South Australia, Australia | 01-Apr-04 |
| WAPr7 | HQ248050 | HQ248023 | *Procladius paludicola* | Brampton Ave, Butler, Western Australia, Australia | 01-Nov-04 |
| Pr186 | HQ248053 | HQ248008 | *Procladius paludicola* | Berwick Springs Lake, Berwick Springs, Victoria, Australia | 20-Dec-02 |
| Pr103 | HQ248068 | HQ247991 | Procladius sp.1 | Nursery Rd, Mt Macedon, Victoria, Australia | 27-Feb-03 |
| Pr12 | HQ248048 | HQ248010 | *Procladius* sp.1 | Red Leap Reserve, Mill Park, Victoria, Australia | 22-Feb-03 |
| Pr274 | HQ248070 | HQ247993 | *Procladius* sp.1 | Hill Lake, Rowville, Victoria, Australia | 23-Feb-03 |
| Pr276 | HQ248069 | HQ247995 | *Procladius* sp.1 | Hill Lake, Rowville, Victoria, Australia | 23-Feb-03 |
| Pr290 | HQ248060 | HQ248013 | *Procladius* sp.1 | Lynbrook Estate Wetlands, Lynbrook Estate, Victoria, Australia | 20-Dec-02 |
| Pr9 | HQ248071 | HQ247994 | *Procladius* sp.1 | Red Leap Reserve, Mill Park, Victoria, Australia | 22-Feb-03 |
| Pr98 | HQ248072 | HQ247992 | *Procladius* sp.1 | Jackson's Creek, Gisborne, Victoria, Australia | 27-Feb-03 |
| Pr41 | HQ247999 | HQ248077 | *Procladius* sp.2 | Hill Lake, Rowville, Victoria, Australia | 23-Feb-03 |
| Pr14 | HQ248000 | HQ248076 | *Procladius* sp.2 | Red Leap Reserve, Mill Park, Victoria, Australia | 22-Feb-03 |
| Pr8 | HQ247998 | HQ248075 | *Procladius* sp.2 | Red Leap Reserve, Mill Park, Victoria, Australia | 22-Feb-03 |
| Pr11 | HQ247996 | HQ248074 | *Procladius* sp.2 | Red Leap Reserve, Mill Park, Victoria, Australia | 22-Feb-03 |
| Pr272 | HQ247997 | HQ248073 | *Procladius* sp.2 | Hill Lake, Rowville, Victoria, Australia | 23-Feb-03 |
| A04Pr11 | HQ248080 | HQ248026 | *Procladius villosimanus* | Queen's Park, Moonee Ponds, Victoria, Australia | 26-Apr-04 |
| A04Pr12 | HQ248083 | HQ248031 | *Procladius villosimanus* | Hartley Rd Reserve, Wonga Park, Victoria, Australia | 21-Apr-04 |
| A04Pr3 | HQ248088 | HQ248032 | *Procladius villosimanus* | Kalparrin Reserve, Greensborough, Victoria, Australia | 19-Apr-04 |
| A04Pr6 | HQ248090 | HQ248034 | *Procladius villosimanus* | Healthy Bay Wetlands, Dandenong North, Victoria, Australia | 07-Apr-04 |
| A04Pr8 | HQ248092 | HQ248036 | *Procladius villosimanus* | Monash Gallery, Wheelers Hill, Victoria, Australia | 20-Apr-04 |
| N04Pr2 | HQ248094 | HQ248028 | *Procladius villosimanus* | Homebush Bay, Sydney, New South Wales, Australia | 01-Apr-03 |
| Pr100 | HQ248067 | HQ247990 | *Procladius villosimanus* | Jackson's Creek, Gisborne, Victoria, Australia | 27-Feb-03 |
| Pr148 | HQ248082 | HQ248038 | *Procladius villosimanus* | Anakie retarding basin, Lara, Victoria, Australia | 27-Feb-03 |
| SAPr2 | HQ248086 | KC750771 | *Procladius villosimanus* | Waste water treatment plant, Bolivar, South Australia, Australia | 22-Jun-04 |
| SAPr3 | HQ248085 | HQ248029 | *Procladius villosimanus* | Waste water treatment plant, Bolivar, South Australia, Australia | 22-Jun-04 |
| WAPr1 | HQ248081 | HQ248027 | *Procladius villosimanus* | Sir McCluster Lake, Iluka Western Australia, Australia | 01-Nov-04 |
| BR08R1 | KC750518 | KC750568 | *Riethia stictoptera* | Barwon River at Pollocksford Rd, Stonehaven, Victoria, Australia | 13-Oct-08 |
| E06R10 | KC750519 |  | *Riethia stictoptera* | Maribyrnong River, Canning St Ford, Avondale Heights Victoria, Australia | 7-Nov-06 |
| M18 | KC750520 | KC750670 | *Riethia stictoptera* | Glynn's Wetland, Warrandyte, Victoria, Australia | 26-Aug-02 |
| S03R1 | KC750671 | KC750773 | *Riethia stictoptera* | Don Reserve, Healesville, Victoria, Australia | 24-Oct-03 |
| S03R16 | KC750521 |  | *Riethia stictoptera* | Red Leap, Mill Park, Victoria, Australia | 08-Oct-03 |
| S03R2 | KC750523 |  | *Riethia stictoptera* | Don Reserve, Healesville, Victoria, Australia | 24-Oct-03 |
| S03R30 | KC750522 | KC750675 | *Riethia stictoptera* | Nursery Rd, Macedon, Victoria, Australia | 22-Oct-03 |
| S03R43 | KC750524 | KC750673 | *Riethia stictoptera* | Healthy Bay Wetlands, Dandenong North, Victoria, Australia | 15-Oct-03 |
| S03R50 | KC750525 | KC750672 | *Riethia stictoptera* | Monbulk RB, Belgrave, Victoria, Australia | 20-Oct-03 |
| S03R62 | KC750526 |  | *Riethia stictoptera* | Hill Lake, Rowville, Victoria, Australia | 15-Oct-03 |
| S03R69 | KC750527 | KC750669 | *Riethia stictoptera* | Glynn's Wetland, Warrandyte, Victoria, Australia | 24-Oct-03 |
| A03T64 | DQ393869 | KC750564 | *Tanytarsus inextentus* | Lagoon Park, Caulder Hwy Macedon, Victoria, Australia | 15-May-03 |
| E06T19 | KC750534 |  | *Tanytarsus inextentus* | Eumemmerring Creek, Belgrave-Hallam Rd, Lysterfield, Victoria, Australia | 14-Nov-06 |
| E06T21 | KC750530 | KC750566 | *Tanytarsus inextentus* | Eumemmerring Creek, Belgrave-Hallam Rd, Lysterfield, Victoria, Australia | 14-Nov-06 |
| S03T224 | DQ393870 | KC750588 | *Tanytarsus inextentus* | Glynn's Wetland, Warrandyte, Victoria, Australia | 24-Oct-03 |
| S03T228 | DQ393851 | KC750565 | *Tanytarsus inextentus* | Glynn's Wetland, Warrandyte, Victoria, Australia | 24-Oct-03 |
| S03T24 | KC750667 | KC750567 | *Tanytarsus inextentus* | Nursery Rd, Macedon, Victoria, Australia | 22-Oct-03 |
| S03T89 |  | KC750666 | *Tanytarsus inextentus* | Crawley Rd Dam, Narre Warren, Victoria, Australia | 13-Oct-03 |
| S04T1 | KC750528 |  | *Tanytarsus inextentus* | Hill Lake, Rowville, Victoria, Australia | 11-Oct-04 |
| S04T150 | KC750533 | KC750563 | *Tanytarsus inextentus* | Ferguson Paddock, Hurstbridge, Victoria, Australia | 15-Oct-04 |
| S04T24 | KC750531 |  | *Tanytarsus inextentus* | Nursery Rd, Macedon, Victoria, Australia | 13-Oct-04 |
| S04T313 | KC750532 | KC750668 | *Tanytarsus inextentus* | Crawley Rd Dam, Narre Warren, Victoria, Australia | 13-Oct-03 |
