# Supplementary material for: Environmental monitoring using next generation sequencing: rapid identification of macroinvertebrate bioindicator species
Source: Front Zool. 2013 Aug 7;10:45. doi: 10.1186/1742-9994-10-45 (PMC3750358; doi:10.1186/1742-9994-10-45)
Supplement: Additional file 2: Table S2 — The number of sequences (reads) generated for each gene from each run (biological replicate), the total for each gene and the total for both genes for each sample. The number of individuals for each species that were present at a site is given as ‘n’. [file 1742-9994-10-45-S2.docx]

**Additional file 2 Table S2. The number of sequences (reads) generated for each gene from each run (biological replicate), the total for each gene and the total for both genes for each sample**. The number of individuals for each species that were present at a site is given as ‘n’

|  |  |  | **Number of 454 sequence generated** | | | | | | |
| --- | --- | --- | --- | --- | --- | --- | --- | --- | --- |
| **Species** | **Sample** | **n** | **COI run 1** | **COI run 2** | **COI**  **total** | **CB run1** | **CB run 2** | **CB all** | **Total** |
| *Ablabesmyia* sp.2 | BR08 | 1 | 13 | 21 | 34 | 6 | 6 | 12 | 46 |
| *Botryocladius* sp.1 | BR08 | 1 | 22 | 43 | 65 | 16 | 15 | 31 | 96 |
| *Chironomus oppositus* | BR08 | 3 | 17 | 18 | 35 | 0 | 0 | 0 | 35 |
| *Cladotanytarsus* sp.C | BR08 | 1 | 0 | 0 | 0 | 2 | 5 | 7 | 7 |
| *Coelopynia* | BR08 | 1 | 16 | 10 | 26 | 0 | 0 | 0 | 26 |
| *Cricotopus* sp.2 | BR08 | 4 | 15 | 14 | 29 | 1 | 1 | 2 | 31 |
| *Parachironomus delinificus* | BR08 | 3 | 159 | 220 | 379 | 29 | 22 | 51 | 430 |
| *Parachironomus* sp.3 | BR08 | 1 | 5 | 2 | 7 | 141 | 176 | 317 | 324 |
| *Polypedilum convexum* | BR08 | 3 | 108 | 103 | 211 | 58 | 63 | 121 | 332 |
| *Procladius paludicola* | BR08 | 11 | 214 | 280 | 494 | 636 | 572 | 1208 | 1702 |
| *Procladius* sp.2 | BR08 | 39 | 1198 | 1499 | 2697 | 954 | 958 | 1912 | 4609 |
| *Riethia stictoptera* | BR08 | 1 | 177 | 194 | 371 | 228 | 314 | 542 | 913 |
| *Chironomus oppositus* | DB09 | 2 | 260 | 300 | 560 | 20 | 23 | 43 | 603 |
| *Cladotanytarsus australomancus* | DB09 | 1 | 5 | 7 | 12 | 3 | 1 | 4 | 16 |
| *Dicrotendipes pseudoconjunctus* | DB09 | 4 | 355 | 360 | 715 | 112 | 172 | 284 | 999 |
| *Dicrotendipes septemmaculatus* | DB09 | 0 | 0 | 0 | 0 | 1 | 0 | 1 | 1 |
| *Kiefferulus intertinctus* | DB09 | 7 | 258 | 323 | 581 | 0 | 0 | 0 | 581 |
| *Paramerina* sp.4 | DB09 | 1 | 24 | 13 | 37 | 20 | 17 | 37 | 74 |
| *Polypedilum* sp.C | DB09 | 43 | 660 | 761 | 1421 | 401 | 545 | 946 | 2367 |
| *Procladius paludicola* | DB09 | 9 | 34 | 39 | 73 | 138 | 138 | 276 | 349 |
| *Procladius* sp.1 | DB09 | 4 | 252 | 282 | 534 | 353 | 495 | 848 | 1382 |
| *Riethia stictoptera* | DB09 | 6 | 149 | 133 | 282 | 266 | 357 | 623 | 905 |
| *Tanytarsus inextentus* | DB09 | 1 | 2 | 8 | 10 | 5 | 7 | 12 | 22 |
| *Chironomus oppositus* | GC09 | 1 | 333 | 339 | 672 | 74 | 188 | 262 | 934 |
| *Cricotopus albitarsis* | GC09 | 4 | 30 | 41 | 71 | 24 | 61 | 85 | 156 |
| *Cricotopus* sp.1 | GC09 | 1 | 2 | 1 | 3 |  |  | 0 | 3 |
| *Cricotopus* sp.2 | GC09 | 19 | 727 | 812 | 1539 | 835 | 1556 | 2391 | 3930 |
| *Dicrotendipes* sp.A | GC09 | 2 | 311 | 386 | 697 | 445 | 735 | 1180 | 1877 |
| *Kiefferulus cornishi* | GC09 | 1 | 288 | 396 | 684 | 2 | 0 | 2 | 686 |
| *Kiefferulus intertinctus* | GC09 | 3 | 518 | 647 | 1165 | 2 | 4 | 6 | 1171 |
| *Paratanytarsus grimmii* | GC09 | 1 | 26 | 27 | 53 | 87 | 168 | 255 | 308 |
| *Procladius* sp.2 | GC09 | 0 | 4 | 0 | 4 | 0 | 0 | 0 | 4 |
| *Chironomus australis* | HW09 | 100 | 1037 | 1271 | 2308 | 1403 | 1607 | 3010 | 5318 |
| *Chironomus cloacalis* | HW09 | 18 | 435 | 539 | 974 | 12 | 12 | 24 | 998 |
| *Chironomus duplex* | HW09 | 6 | 153 | 252 | 405 | 0 | 0 | 0 | 405 |
| *Chironomus februarius* | HW09 | 22 | 504 | 568 | 1072 | 9 | 23 | 32 | 1104 |
| *Dicrotendipes pseudoconjunctus* | HW09 | 3 | 42 | 52 | 94 | 2 | 4 | 6 | 100 |
| *Dicrotendipes septemmaculatus* | HW09 | 1 | 11 | 6 | 17 | 0 | 0 | 0 | 17 |
| *Kiefferulus intertinctus* | HW09 | 1 | 19 | 11 | 30 | 0 | 0 | 0 | 30 |
| *Paratrichocladius* sp.1 | HW09 | 1 | 2 | 4 | 6 | 0 | 0 | 0 | 6 |
| *Polypedilum nubifer* | HW09 | 9 | 196 | 195 | 391 | 7 | 3 | 10 | 401 |
| *Polypedilum* sp.C | HW09 | 0 | 0 | 2 | 2 | 0 | 0 | 0 | 2 |
| *Procladius paludicola* | HW09 | 1 | 9 | 4 | 13 | 4 | 1 | 5 | 18 |
| *Procladius villosimanus* | HW09 | 5 | 258 | 284 | 542 | 55 | 67 | 122 | 664 |
| *Chironomus australis* | LE09 | 1 | 6 | 6 | 12 | 155 | 151 | 306 | 318 |
| *Chironomus cloacalis* | LE09 | 11 | 421 | 397 | 818 | 380 | 382 | 762 | 1580 |
| *Chironomus duplex* | LE09 | 3 | 80 | 68 | 148 | 0 | 1 | 1 | 149 |
| *Chironomus februarius* | LE09 | 6 | 146 | 152 | 298 | 257 | 266 | 523 | 821 |
| *Chironomus oppositus* | LE09 | 57 | 2123 | 1990 | 4113 | 1031 | 1053 | 2084 | 6197 |
| *Chironomus tepperi* | LE09 | 3 | 16 | 26 | 42 | 49 | 75 | 124 | 166 |
| *Cladopelma* sp.1 | LE09 | 0 | 0 | 0 | 0 | 4 | 0 | 4 | 4 |
| *Coelopynia* | LE09 | 0 | 0 | 0 | 0 | 0 | 1 | 1 | 1 |
| *Dicrotendipes pseudoconjunctus* | LE09 | 6 | 96 | 111 | 207 | 141 | 131 | 272 | 479 |
| *Kiefferulus intertinctus* | LE09 | 2 | 16 | 16 | 32 | 0 | 0 | 0 | 32 |
| *Kiefferulus martini* | LE09 | 1 | 11 | 30 | 41 | 74 | 53 | 127 | 168 |
| *Parachironomus delinificus* | LE09 | 1 | 2 | 1 | 3 | 4 | 3 | 7 | 10 |
| *Paralimnophyes* sp.1 | LE09 | 2 | 4 | 5 | 9 | 29 | 31 | 60 | 69 |
| *Paratanytarsus grimmii* | LE09 | 3 | 28 | 29 | 57 | 107 | 100 | 207 | 264 |
| *Procladius villosimanus* | LE09 | 2 | 36 | 20 | 56 | 120 | 109 | 229 | 285 |
| *Chironomus australis* | MC09 | 0 |  |  | 0 | 1 | 1 | 2 | 2 |
| *Chironomus oppositus* | MC09 | 2 | 346 | 403 | 749 | 21 | 45 | 66 | 815 |
| *Cladopelma* sp.1 | MC09 | 2 | 335 | 376 | 711 | 64 | 94 | 158 | 869 |
| *Cladotanytarsus australomancus* | MC09 | 14 | 718 | 870 | 1588 | 1129 | 1522 | 2651 | 4239 |
| *Cricotopus* sp.2 | MC09 | 6 | 325 | 373 | 698 | 147 | 164 | 311 | 1009 |
| *Dicrotendipes pseudoconjunctus* | MC09 | 1 | 324 | 458 | 782 | 124 | 158 | 282 | 1064 |
| *Polypedilum* sp.C | MC09 | 2 | 239 | 370 | 609 | 132 | 188 | 320 | 929 |
| *Polypedilum* sp.E | MC09 | 1 | 55 | 78 | 133 | 73 | 78 | 151 | 284 |
| *Procladius paludicola* | MC09 | 0 |  |  | 0 | 0 | 1 | 1 | 1 |
| *Chironomus australis* | ME09 | 18 | 89 | 161 | 250 | 887 | 1233 | 2120 | 2370 |
| *Chironomus cloacalis* | ME09 | 1 | 25 | 46 | 71 | 4 | 4 | 8 | 79 |
| *Chironomus duplex* | ME09 | 1 | 108 | 145 | 253 | 0 | 0 | 0 | 253 |
| *Corynoneura scutellata* | ME09 | 2 | 8 | 3 | 11 | 3 | 2 | 5 | 16 |
| *Cricotopus annuliventris* | ME09 | 1 | 15 | 11 | 26 | 1 |  | 1 | 27 |
| *Dicrotendipes pseudoconjunctus* | ME09 | 21 | 1326 | 1883 | 3209 | 663 | 852 | 1515 | 4724 |
| *Kiefferulus cornishi* | ME09 | 1 | 9 | 20 | 29 | 0 | 0 | 0 | 29 |
| *Kiefferulus intertinctus* | ME09 | 1 | 91 | 86 | 177 | 0 | 0 | 0 | 177 |
| *Paralimnophyes* sp.1 | ME09 | 2 | 9 | 5 | 14 | 0 | 13 | 13 | 27 |
| *Paratanytarsus* sp.D | ME09 | 1 | 1 | 5 | 6 | 0 | 1 | 1 | 7 |
| *Procladius villosimanus* | ME09 | 2 | 310 | 387 | 697 | 287 | 408 | 695 | 1392 |
| *Chironomus australis* | RL09 | 1 | 16 | 30 | 46 | 130 | 218 | 348 | 394 |
| *Chironomus cloacalis* | RL09 | 1 | 5 | 4 | 9 | 12 | 2 | 14 | 23 |
| *Chironomus duplex* | RL09 | 3 | 107 | 118 | 225 | 0 | 0 | 0 | 225 |
| *Chironomus februarius* | RL09 | 20 | 238 | 334 | 572 | 98 | 191 | 289 | 861 |
| *Chironomus oppositus* | RL09 | 22 | 255 | 319 | 574 | 83 | 123 | 206 | 780 |
| *Chironomus pseudoppositus* | RL09 | 3 | 42 | 53 | 95 | 0 | 1 | 1 | 96 |
| *Chironomus tepperi* | RL09 | 1 | 1 | 3 | 4 | 1 | 1 | 2 | 6 |
| *Cladopelma* sp.2 | RL09 | 8 | 58 | 38 | 96 | 72 | 124 | 196 | 292 |
| *Dicrotendipes pseudoconjunctus* | RL09 | 7 | 56 | 56 | 112 | 44 | 42 | 86 | 198 |
| *Dicrotendipes* sp.4 | RL09 | 1 | 2 | 2 | 4 | 6 | 8 | 14 | 18 |
| *Kiefferulus martini* | RL09 | 1 | 3 | 10 | 13 | 0 | 5 | 5 | 18 |
| *Microchironomus forcipatus* | RL09 | 1 | 0 | 2 | 2 | 0 | 5 | 5 | 7 |
| *Procladius villosimanus* | RL09 | 30 | 1381 | 1882 | 3263 | 1378 | 2019 | 3397 | 6660 |
| *Tanytarsus inextentus* | RL09 | 1 | 0 | 0 | 0 | 1 | 4 | 5 | 5 |
| *Chironomus australis* | SK09 | 1 | 0 | 0 | 0 | 4 | 4 | 8 | 8 |
| *Chironomus februarius* | SK09 | 0 | 0 | 0 | 0 | 0 | 1 | 1 | 1 |
| *Chironomus oppositus* | SK09 | 31 | 1570 | 1722 | 3292 | 568 | 943 | 1511 | 4803 |
| *Corynoneura scutellata* | SK09 | 1 |  |  |  | 2 | 1 | 3 | 3 |
| *Dicrotendipes pseudoconjunctus* | SK09 | 1 | 49 | 29 | 78 | 219 | 282 | 501 | 579 |
| *Dicrotendipes* sp.4 | SK09 | 2 | 40 | 39 | 79 | 398 | 418 | 816 | 895 |
| *Kiefferulus ‘cornishi’* | SK09 | 4 | 238 | 274 | 512 | 1 | 3 | 4 | 516 |
| *Kiefferulus intertinctus* | SK09 | 5 | 872 | 846 | 1718 | 7 | 12 | 19 | 1737 |
| *Paratanytarsus grimmii* | SK09 | 5 | 105 | 100 | 205 | 559 | 719 | 1278 | 1483 |
| *Paratrichocladius* sp.2 | SK09 | 1 | 13 | 6 | 19 | 98 | 94 | 192 | 211 |
| *Chironomus australis* | UK09 | 71 | 968 | 1036 | 2004 | 2012 | 1503 | 3515 | 5519 |
| *Chironomus cloacalis* | UK09 | 5 | 242 | 253 | 495 | 11 | 5 | 16 | 511 |
| *Chironomus duplex* | UK09 | 6 | 1071 | 1256 | 2327 | 0 | 0 |  | 2327 |
| *Chironomus februarius* | UK09 | 2 | 31 | 36 | 67 | 10 | 5 | 15 | 82 |
| *Chironomus nepeanensis* | UK09 | 1 | 80 | 125 | 205 | 46 | 47 | 93 | 298 |
| *Chironomus oppositus* | UK09 | 1 | 0 | 0 | 0 | 0 | 0 | 0 | 0 |
| *Cladopelma* sp.1 | UK09 | 1 | 4 | 6 | 10 | 0 | 0 | 0 | 10 |
| *Kiefferulus cornishi* | UK09 | 1 | 34 | 41 | 75 | 0 | 0 | 0 | 75 |
| *Kiefferulus intertinctus* | UK09 | 3 | 114 | 122 | 236 | 0 | 0 | 0 | 236 |
| *Paralimnophyes* sp.1 | UK09 | 0 | 2 | 0 | 2 | 0 | 0 | 0 | 2 |
| *Polypedilum nubifer* | UK09 | 1 | 16 | 45 | 61 | 1 | 1 | 2 | 63 |
| *Polypedilum* sp.E | UK09 | 1 | 3 | 4 | 7 | 2 | 0 | 2 | 9 |
| *Procladius paludicola* | UK09 | 2 | 11 | 28 | 39 | 29 | 27 | 56 | 95 |
