# Supplementary material for: Environmental monitoring using next generation sequencing: rapid identification of macroinvertebrate bioindicator species
Source: Front Zool. 2013 Aug 7;10:45. doi: 10.1186/1742-9994-10-45 (PMC3750358; doi:10.1186/1742-9994-10-45)
Supplement: Additional file 3: Figure S1 — PCR primer map and amplicon lengths. Primer position and PCR fragment lengths for a) COI and b) CytB used in DNA reference database construction and 454 pyrosequencing. Primer positions are indicated in blue. [file 1742-9994-10-45-S3.docx]

**Additional file 3 Figure S1. PCR primer map and amplicon lengths**. Primer position and PCR fragment lengths (for a) COI and b) CytB used in DNA reference database construction and 454 pyrosequencing. Primer positions are indicated in blue.

a)

509bp

709bp

LCOI/ 911

COI A for

HCOI/912

1

1539

b)

Variable 810-850bp

619bp

393bp

CB1

CB322R

T-N-S1
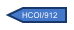
1

CB549R
