# Supplementary material for: Environmental monitoring using next generation sequencing: rapid identification of macroinvertebrate bioindicator species
Source: Front Zool. 2013 Aug 7;10:45. doi: 10.1186/1742-9994-10-45 (PMC3750358; doi:10.1186/1742-9994-10-45)
Supplement: Additional file 4: Table S3 — PCR fusion primers used to amplify sample for 454 pyrosequencing. Site code refers to which site each primer pair was used. Forward primers contain 454 adapter A (designated as ‘A’ at the end of the primer name) and reverse primers contain 454 adapter B (designated as ‘B’ at the end of the primer name), Each primer contains a different MID as indicated with parentheses. [file 1742-9994-10-45-S4.docx]

**Additional file 4 Table S3.** **PCR fusion primers used to amplify sample for 454 pyrosequencing**. Site code refers to which site each primer pair was used. Forward primers contain 454 adapter A (designated as ‘A’ at the end of the primer name) and reverse primers contain 454 adapter B (designated as ‘B’ at the end of the primer name), Each primer contains a different MID as indicates with parentheses.

| **Site code** | **Primer name** | **Sequence (5’ to 3’)** |
| --- | --- | --- |
| BR08 | 60 UT 1(MID1)A | CGTATCGCCTCCCTCGCGCCATCAGACGAGTGCGTCAGGACCAGGgTAcgGTG |
|  | 60 UT 1(MID2)B | CTATGCGCCTTGCCAGCCCGCTCAGACGCTCGACAcGCAGagAGgctccgTG |
| DB09 | 60 UT 2(MID3)A | CGTATCGCCTCCCTCGCGCCATCAGAGACGCACTCCAGGACCAGGgTAcgGTG |
|  | 60 UT 2(MID4)B | CTATGCGCCTTGCCAGCCCGCTCAGAGCACTGTAGcGCAGagAGgctccgTG |
| GC09 | 60 UT 3(MID5)A | CGTATCGCCTCCCTCGCGCCATCAGATCAGACACGCAGGACCAGGgTAcgGTG |
|  | 60 UT 3(MID8)B | CTATGCGCCTTGCCAGCCCGCTCAGCTCGCGTGTCcGCAGagAGgctccgTG |
| HW09 | 60 UT 4(MID11)B | CTATGCGCCTTGCCAGCCCGCTCAGTGATACGTCTcGCAGagAGgctccgTG |
|  | 60 UT 4(MID20)A | CGTATCGCCTCCCTCGCGCCATCAGACGACTACAGCAGGACCAGGgTAcgGTG |
| LE09 | 60 UT 5(MID13)A | CGTATCGCCTCCCTCGCGCCATCAGCATAGTAGTGCAGGACCAGGgTAcgGTG |
|  | 60 UT 5(MID14)B | CTATGCGCCTTGCCAGCCCGCTCAGCGAGAGATACcGCAGagAGgctccgTG |
| MC09 | 60 UT 6(MID15)A | CGTATCGCCTCCCTCGCGCCATCAGATACGACGTACAGGACCAGGgTAcgGTG |
|  | 60 UT 6(MID16)B | CTATGCGCCTTGCCAGCCCGCTCAGTCACGTACTAcGCAGagAGgctccgTG |
| ME09 | 60 UT 7(MID19)A | CGTATCGCCTCCCTCGCGCCATCAGTGTACTACTCCAGGACCAGGgTAcgGTG |
|  | 60 UT 7(MID28)B | CTATGCGCCTTGCCAGCCCGCTCAGACTACTATGTcGCAGagAGgctccgTG |
| RL09 | 60 UT 9(MID23)A | CGTATCGCCTCCCTCGCGCCATCAGTACTCTCGTGCAGGACCAGGgTAcgGTG |
|  | 60 UT 9(MID34)B | CTATGCGCCTTGCCAGCCCGCTCAGCACGCTACGTcGCAGagAGgctccgTG |
| SK09 | 60 UT 10(MID25)A | CGTATCGCCTCCCTCGCGCCATCAGTCGTCGCTCGCAGGACCAGGgTAcgGTG |
|  | 60 UT 10(MID32)B | CTATGCGCCTTGCCAGCCCGCTCAGAGTACGCTATcGCAGagAGgctccgTG |
| UK09 | 60 UT 11(MID31)A | CGTATCGCCTCCCTCGCGCCATCAGAGCGTCGTCTCAGGACCAGGgTAcgGTG |
|  | 60 UT 11(MID38)B | CTATGCGCCTTGCCAGCCCGCTCAGTACACGTGATcGCAGagAGgctccgTG |
| Control Cc1 (*Chironomus cloacalis*) | 60 UT 12(MID33)A | CGTATCGCCTCCCTCGCGCCATCAGATAGAGTACTCAGGACCAGGgTAcgGTG |
|  | 60 UT 12(MID39)B | CTATGCGCCTTGCCAGCCCGCTCAGTACAGATCGTcGCAGagAGgctccgTG |
| Control Cf1 (*Chironomus februarius*) | 60 UT 13(MID35)A | CGTATCGCCTCCCTCGCGCCATCAGCAGTAGACGTCAGGACCAGGgTAcgGTG |
|  | 60 UT 13(MID40)B | CTATGCGCCTTGCCAGCCCGCTCAGTACGCTGTCTcGCAGagAGgctccgTG |
| Control Cf2 (*Chironomus februarius*) | 60UT 14 MID37A | CGTATCGCCTCCCTCGCGCCATCAGTACACACACTCAGGACCAGGGTACGGTG |
|  | 60UT 14 MID41B | CTATGCGCCTTGCCAGCCCGCTCAGTAGTGTAGATcGCAGAGAGgctccgTG |
